# The Janus Face of *Aspergillus* in Fish Aquaculture: From Pathogenic Threat to Functional Feed Additive

**DOI:** 10.3390/vetsci13080737

**Published:** 2026-07-24

**Authors:** Michelyne Haroun, Christophe Tratrat, Roshmon Thomas Mathew, Muhammad Munir, Mohamed Shawky, Ouda Nasser Aldakhilallah, Mohamed Ashour, Sahar Mohamed Ibrahim, Athina Geronikaki

**Affiliations:** 1 Fish Resources Research Center, King Faisal University, Al-Ahsa 31982, Saudi Arabia; rmathew@kfu.edu.sa (R.T.M.); oaldakhilalla@kfu.edu.sa (O.N.A.); mashour@kfu.edu.sa (M.A.); 2 Department of Pharmaceutical Sciences, College of Clinical Pharmacy, King Faisal University, Al-Ahsa 31982, Saudi Arabia; ctratrat@kfu.edu.sa; 3 Date Palm Research Center of Excellence, King Faisal University, Al-Ahsa 31982, Saudi Arabia; 4 Avian Research Center, King Faisal University, Al-Ahsa 31982, Saudi Arabia; mabdelmoaty@kfu.edu.sa; 5 Department of Pharmacy Practice, College of Clinical Pharmacy, King Faisal University, Al-Ahsa 31982, Saudi Arabia; smibrahim@kfu.edu.sa; 6 Department of Pharmaceutical Chemistry, School of Pharmacy, Aristotle University of Thessaloniki, 54124 Thessaloniki, Greece; geronik@pharm.auth.gr

**Keywords:** *Aspergillus*, fish aquaculture, aspergillosis, aflatoxins, fungal probiotics, solid-state fermentation, mycotoxins, *Oreochromis niloticus*, functional feed, One Health

## Abstract

The dramatic rise in global aquaculture has brought renewed focus on filamentous fungi impacting fish health and aquafeeds. The fungal genus *Aspergillus* has a Janus-like role in aquaculture. On the pathogenic side, opportunistic pathogens like *A. flavus*, *A. niger* and *A. fumigatus* cause outbreaks of aspergillosis, with granulomatous lesions and significant mortality, especially in fish with impaired immunity and/or poor environmental conditions. Toxigenic strains of *A. flavus* and *A. parasiticus* also contaminate aquafeeds with aflatoxins, some of the most toxic natural hepatotoxins and carcinogens, which have been shown to affect growth, immunity and aflatoxin residues in edible fish fillets. On the positive side, non-toxigenic strains of *A. niger*, *A. oryzae* and *A. awamori* are increasingly used as fungal probiotics, solid-state fermenters to upgrade plant protein feed supplements, and as sources of industrially useful hydrolytic enzymes, secondary metabolites and antifungal compounds. Synthesizing evidence from over 90 peer-reviewed studies, this narrative review elucidates how species, strain, and rearing context jointly determine whether *Aspergillus* behaves as a pathogen or as a functional additive. We highlight knowledge gaps, offer an interpretative model and suggest practical strategies to limit risks and leverage the biotechnological uses of *Aspergillus* for sustainable aquaculture. The novelty of this review lies in integrating, within a single finfish-focused framework, the three usually separate faces of *Aspergillus*—pathogen, aflatoxin producer, and functional feed additive—and in translating this dual nature into practical, strain-level guidance for aquaculture.

## Simple Summary

Fish and shellfish aquaculture is the fastest-growing animal food production system in the world, and its future relies on maintaining the health of farmed fish and producing quality feed. The mold genus *Aspergillus* is an unexpected double-edged sword among microorganisms that affect aquaculture. Certain species of the fungus are pathogens of fish or they contaminate fish feed with toxins (aflatoxins) that harm the liver, stunt fish growth and pose risks for human health. Yet, other species of the same genus are added as feed supplements: they are probiotics, ferment plant feedstuffs to increase digestibility and produce enzymes. This review provides a synthesis of our current knowledge of both sides of *Aspergillus* in fish aquaculture, helping farmers, veterinarians and researchers to understand when and how this fungus is a threat and how they can harness it as an ally.

## 1. Introduction

Aquaculture is the fastest-growing animal food production system and more than 50% of aquatic animal food is now produced in aquaculture systems [1]. This is in line with aquaculture intensification, increased fish density in aquaculture ponds and aquaculture production cycles, and replacement of fishmeal with plant-based ingredients. Egypt is one of the significant aquaculture-producing countries with tilapia as the most intensively aquacultured species, and recent surveys have revealed that there is a need for improved infrastructure, technology and species-specific aquaculture systems to enhance sustainability and productivity of the aquaculture production systems [2]. All of these developments provide opportunities for microbial pathogens, especially filamentous fungi, which grow on feed ingredients and stressed fish. Of special interest is the genus *Aspergillus*, because of its diversity, ubiquity and apparent paradox in aquaculture. *Aspergillus* is a genus of over 350 described filamentous ascomycete fungi found in the environment in soil, air, water and on decaying organic matter [3]. This adaptability to different environments is reflected in a variety of interactions with animal hosts. Some *Aspergillus* species are opportunistic pathogens: *A. fumigatus*, *A. flavus*, and *A. niger* are recognized pathogens of fish, causing aspergillosis in several commercially important aquaculture species [4]. Toxigenic strains of *A. flavus* and *A. parasiticus* are also the principal natural producers of aflatoxins, which are among the most hepatotoxic and carcinogenic compounds ever characterized. Other *Aspergillus* species, by contrast, have been domesticated by human industries for millennia and are listed as Generally Recognized as Safe (GRAS) by the U.S. Food and Drug Administration. *A. oryzae* has been used for centuries in East Asian food fermentation, while *A. niger* and *A. awamori* are the workhorses of modern enzyme manufacturing, organic acid production, and, more recently, of fungal probiotics developed specifically for aquaculture [5].

Within fish aquaculture, this duality is not abstract. The same genus that triggers mortality outbreaks [6], with *Aspergillus* also documented as a pathogen of carp [7,8], also provides fermentation starters that upgrade soybean, rapeseed, and olive cake into more digestible, more sustainable feed ingredients [9,10] [11]. Fish farmers report aspergillosis lesions in diseased fish [8,12,13,14], while researchers simultaneously demonstrate improved growth, immune responses, and disease resistance in tilapia, carp, and sea bream fed diets supplemented with *A. oryzae* or *A. niger* products [15,16,17,18]. This apparent paradox is not a contradiction in this evidence but an expression of the biology of the genus: pathogenic, toxic and probiotic traits of *Aspergillus* species are not uniformly distributed and are strain- and context-specific.

A number of excellent reviews have addressed related topics, such as fungi in aquaculture generally [5] [19], *A. niger* in shrimp in particular [20], emerging mycotoxins in aquafeed [21,22,23,24], aflatoxicosis in fish [25,26], marine-derived *Aspergillus* as a source of antimicrobials [27,28], phytobiotics in aquaculture [29], and alternatives to antibiotics for sustainable aquaculture [30]. Yet, there has been no attempt to connect the “dark” and “light” sides of *Aspergillus* for finfish aquaculture. Existing reviews have addressed these dimensions in isolation, covering either fungal disease in aquaculture, or mycotoxins in aquafeeds, or functional feed additives, whereas none has integrated the pathogenic, aflatoxigenic and beneficial faces of *Aspergillus* within a single, finfish-focused One Health framework. The present narrative review fills this gap by examining the role of *Aspergillus* in finfish aquaculture and reviewing the recent literature on the pathogenic and beneficial faces of *Aspergillus*, particularly over the past 15 years. This study is structured around three content pillars: *Aspergillus* as a direct pathogen, *Aspergillus* as an indirect pathogen through aflatoxin contamination of feeds, *Aspergillus* as a beneficial feed additive, and an integrated discussion about the paradox, knowledge gaps and recommendations for sustainable aquaculture.

This narrative review was based on a structured literature search of PubMed, Scopus, Web of Science and Google Scholar, combining the terms “Aspergillus”, “aspergillosis”, “aflatoxin”, “mycotoxin”, “aquaculture”, “fish”, “tilapia”/“Oreochromis”, “carp”, “probiotic” and “solid-state fermentation”. Priority was given to peer-reviewed, English-language studies published between 2010 and 2026 and directly relevant to finfish aquaculture, while a small number of seminal earlier works were retained for historical and taxonomic context. Studies addressing exclusively human-clinical or non-aquaculture systems were included only where required to establish species-level biological traits (e.g., conidiophore morphology and biofilm formation) that are conserved regardless of isolate origin. As a narrative rather than systematic review, no formal PRISMA protocol or quantitative meta-analysis was applied; studies were selected for their relevance to the pathogenic–beneficial duality of *Aspergillus* in fish aquaculture. Throughout this review, every section is anchored to finfish aquaculture; general mycological, clinical or industrial aspects of *Aspergillus* are discussed only where they bear directly on fish health or aquafeed.

## 2. *Aspergillus* as a Direct Pathogen: Fish Aspergillosis

### 2.1. Historical Background

*Aspergillus* has been known as a primary or opportunistic pathogen of farmed fish since early reports of visceral and cutaneous mycoses in freshwater fish species. Notable early compilations by Olufemi and others collated isolated case studies, confirming *Aspergillus* as a fungal pathogen that should be considered for emerging aquaculture industries [31]. For several decades, the investigation of fungal diseases in fish was dominated by oomycetes (particularly *Saprolegnia* spp.) which, although superficially resembling fungi, are phylogenetically different and more visible on the fish surface than true fungi [32,33]. As a consequence, fish aspergillosis has likely been under-reported in the past, and many early cases were likely to have been misidentified or diagnosed only as “mycotic infection” without species-level identification. Surging interest in *Aspergillus* emerged from the 2000s, spurred by two interacting factors: the adoption of tropical and subtropical aquaculture in geographical areas where the combination of warm water and high humidity is optimal for the development of *Aspergillus* [34], and the widespread adoption of molecular techniques, allowing clear species-level identification of isolates from diseased fish [13,35]. The inclusion of *A. fumigatus* in the WHO list of priority fungal pathogens in human health [36] has also increased One Health interest in this species, which has implications for fish health and aquaculture biosecurity, and alongside the traditional focus on bacterial and viral fish pathogens, fungal pathogens such as *Aspergillus* are now also recognized as being relevant to aquaculture biosecurity.

### 2.2. Aspergillus Species Involved in Fish Aspergillosis

The most common *Aspergillus* species reported from diseased cultured fish are *A. flavus*, *A. niger*, *A. fumigatus*, *A. terreus*, and *A. sydowii* [7,13,14,34,35]. *A. flavus* stands out as the most common species in reports on Nile tilapia in Egypt and Saudi Arabia [12,13,37,38], and is involved in both natural infections and experimentally challenged fish [34,35,37,38]. However, in Indonesia, Arumugam et al. found *A. fumigatus* to be the most common (42%) isolate, while *A. flavus* only accounted for 4% of the isolates [14]. *A. niger* is an additional isolate, which has been reported as pathogenic in freshwater fish, such as carp and catfish [7,35]. *A. fumigatus* and *A. sydowii*, are less commonly reported but cause severe systemic disease in experimentally challenged freshwater fish [7]. Table 1 provides a summary of the main reports of *Aspergillus* species isolated from cultured fish.

#### 2.2.1. Morphological and Microscopic Identification

**Figure 1:**
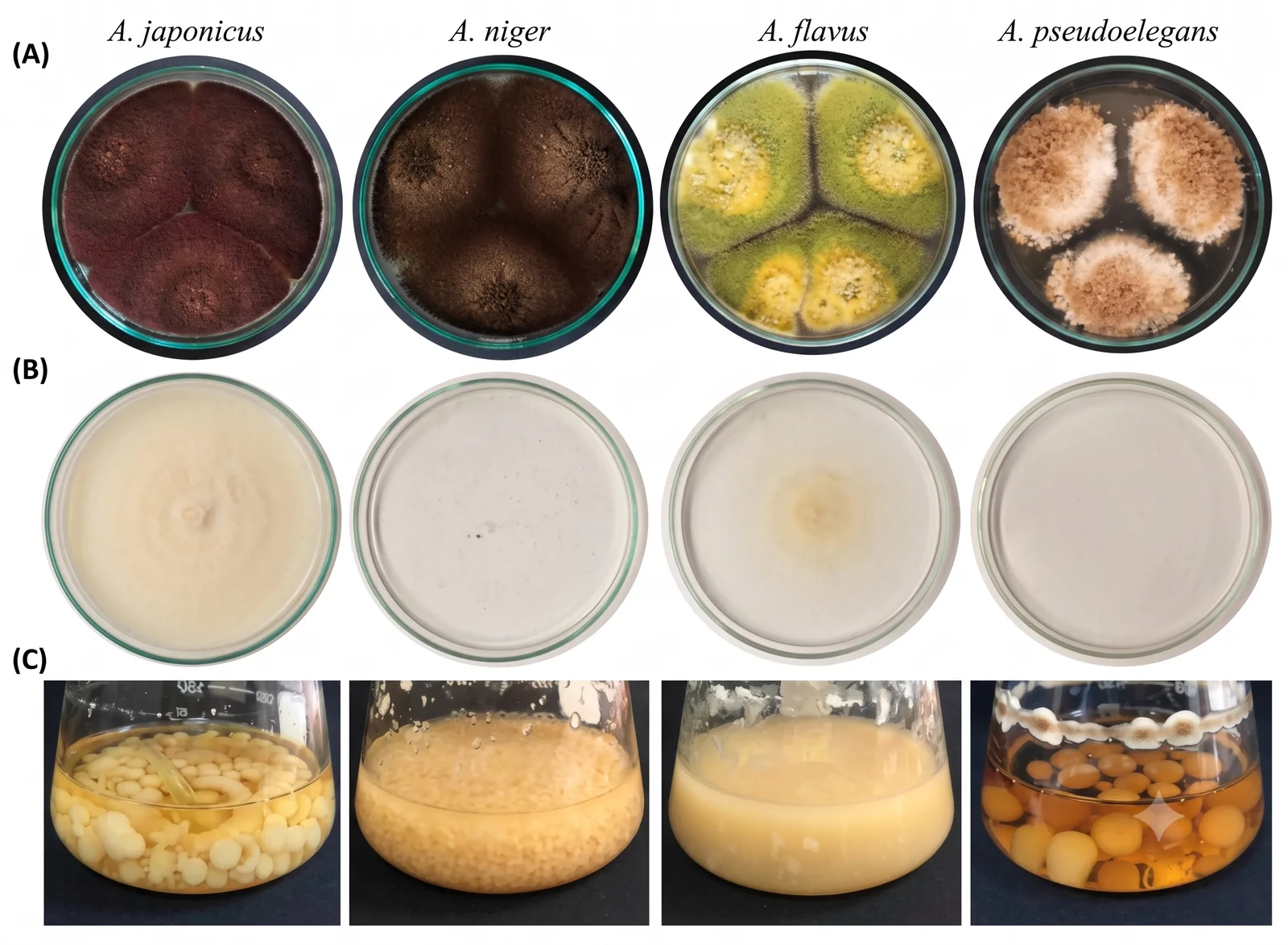
Morphological characterization of four *Aspergillus* species commonly encountered in aquaculture feed contamination: *A. japonicus*, *A. niger*, *A. flavus*, and *A. pseudoelegans*. (**A**) Three-point inoculation colonies on malt extract agar (MEA) after 7 days at 28 °C; (**B**) glass lids of dishes showing characteristic spore dust; (**C**) mold-like growth in malt extract broth (MEB). Species-level morphology is a universal trait independent of the substrate of origin, making these descriptions directly applicable to aquafeed isolates. Reproduced from Atallah et al., J Fungi 2022, 8(6), 626 under CC BY 4.0 license [44].

Correct identification of *Aspergillus* isolates of fish or aquafeeds can be achieved through a combination of macroscopic colony morphology, sporulation cycle, and microscopic characteristics of the reproductive structures [3]. At the macroscopic scale, the four primary species of importance to aquaculture, *A. flavus*, *A. niger*, *A. fumigatus*, and *A. pseudoelegans*, have characteristic colony colors and textures on common mycological media like malt extract agar (MEA). Characteristic appearances of *A. flavus* colonies include yellow-green with a floccose texture, *A. niger* colony is black to dark brown with excessive black conidial heads, and *A. japonicus* colony presents a coffee-brown reverse (Figure 1A) [44]. The sporulation intensity and spore liberation patterns, which can be observed on the glass lids of the Petri dishes as a “spore dust” (Figure 1B), differ across species and depend on the type of seriation of conidiophores [44]. Malt extract broth (MEB) in liquid culture forms characteristic pellet or film morphologies (Figure 1C), which can be utilized in diagnostic laboratories [44].

**Figure 2:**
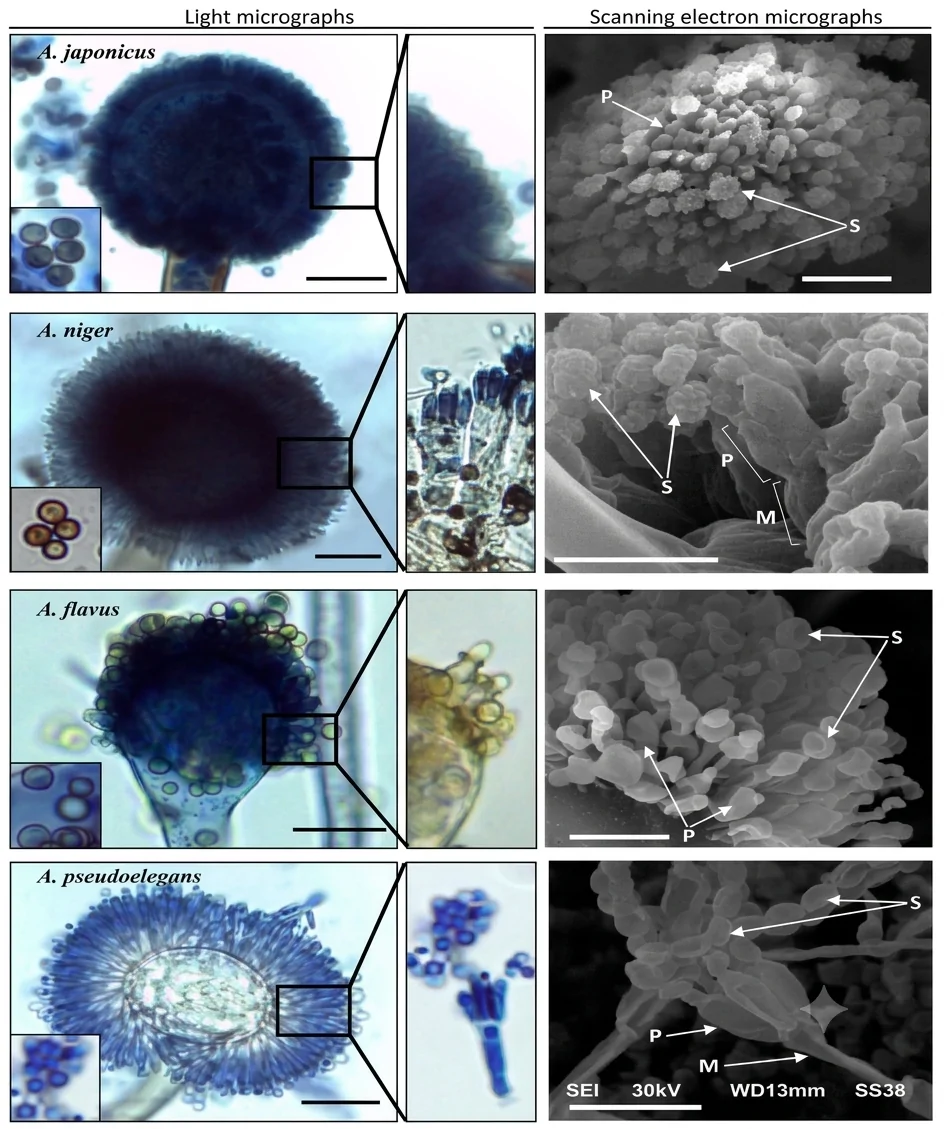
Light (**left column**) and scanning electron micrographs (**right column**) of conidiophores of four *Aspergillus* species. Spores (S) are visible in the small inset panels; phialides (P) and seriation types appear at higher magnification; metulae (M) are indicated in biseriate fungal heads (e.g., *A. niger*, *A. pseudoelegans*). Scale bar: 10 μm in both light and electron micrographs. These microscopic characteristics allow species-level identification of aquaculture isolates. Reproduced from Atallah et al., J Fungi 2022, 8(6), 626 under CC BY 4.0 license [44].

At the microscopic level, light microscopy and scanning electron microscopy (SEM) reveal species-specific conidiophore architectures that remain the gold standard for morphological identification (Figure 2) [3]. Each *Aspergillus* species produces a characteristic conidial head composed of a swollen vesicle bearing phialides (P), conidiogenous cells that produce chains of asexual spores (S). In biseriate species such as *A. niger* and *A. pseudoelegans*, an additional layer of supporting cells called metulae (M) is intercalated between the vesicle and the phialides. Uniseriate species (*A. japonicus*, *A. flavus*) lack metulae [3,44]. Spore surface ornamentation, smooth versus rough, spherical versus ellipsoidal, is best resolved by SEM and provides further discriminating characters [44]. These microscopic features are species-level traits and remain valid regardless of whether the isolate originates from plant biocontrol systems, clinical specimens, or aquafeed contamination.

#### 2.2.2. Biofilm Formation and Environmental Persistence

**Figure 3:**
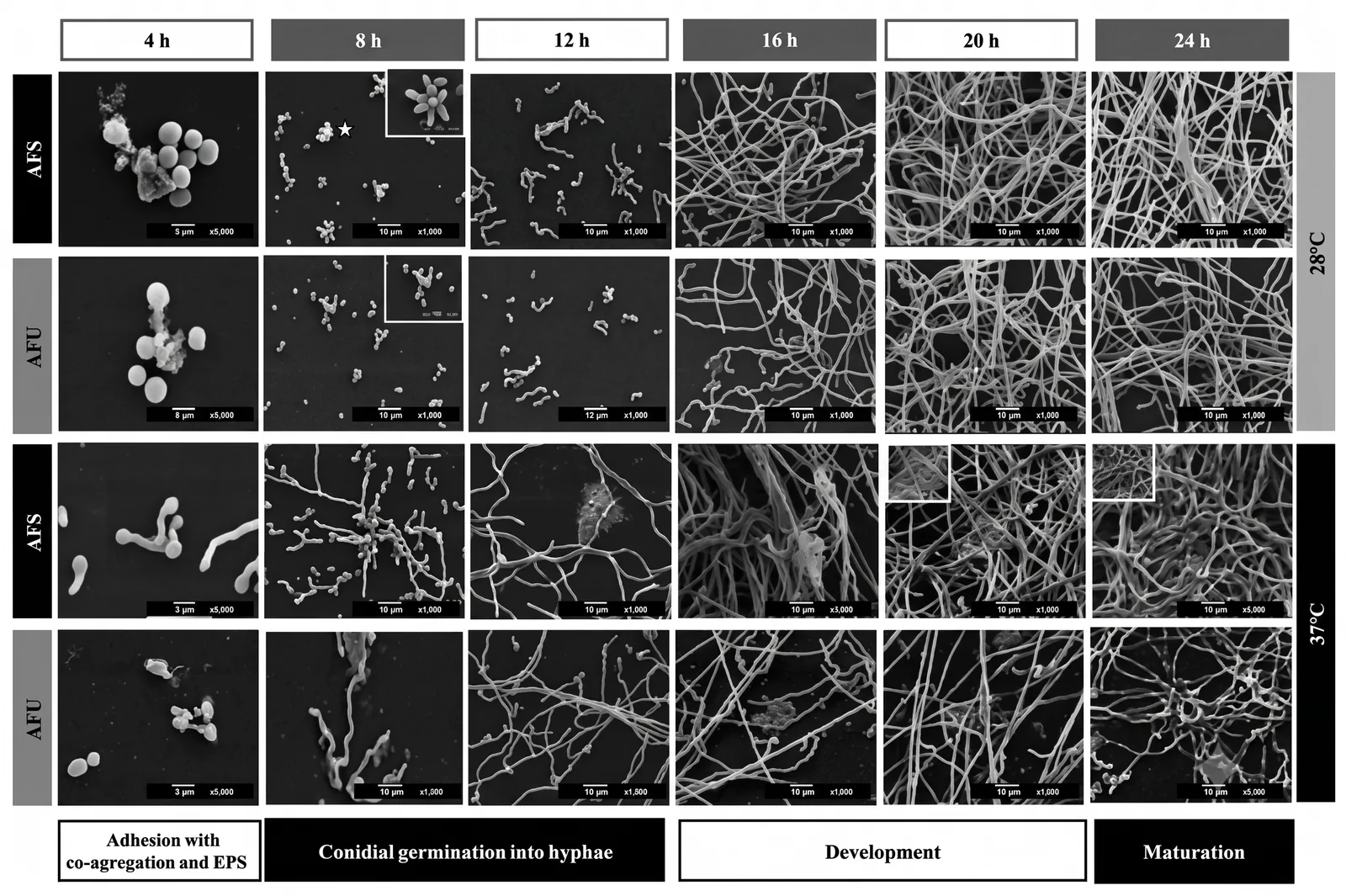
Stages of *Aspergillus fumigatus* biofilm formation described by scanning electron microscopy (SEM), comparing clinical (AFU) and soil (AFS) isolates at 28 °C and 37 °C. Inoculum concentration: 1 × 10^6^ conidia/mL. (i) Adhesion with co-aggregation and exopolymeric substance (EPS) production at 4 h; (ii) conidial germination into hyphae (8–12 h) and development (16–20 h); (iii) mature biofilm (24 h). Although *Aspergillus* biofilms have been primarily characterized in clinical human isolates, biofilm formation is a species-level trait of *A. fumigatus* relevant to its persistence in aquaculture facilities, including tank walls, recirculating system biofilters, and feed storage surfaces. Reproduced from González-Ramírez et al., BMC Microbiology 2016, 16, 243 under CC BY 4.0 license [45].

Beyond vegetative mycelial growth, *Aspergillus* species, and *A. fumigatus* in particular, are able to organize into structured multicellular communities known as biofilms, which display emergent properties such as increased resistance to antifungal drugs, immune evasion, and robust adhesion to biotic and abiotic surfaces. Although biofilm formation by *A. fumigatus* has so far been characterized mostly in the context of clinical human isolates (invasive pulmonary aspergillosis, aspergilloma, medical devices) and soil isolates, it is increasingly recognized as a species-level biological property that is directly relevant to aquaculture because it governs environmental persistence on tank walls, biofilter media in recirculating aquaculture systems (RAS), storage silo surfaces, and pipework [45]. Biofilm development follows a stereotyped sequence of four stages: (i) adhesion with cell co-aggregation and secretion of exopolymeric substance (EPS) at 4 h; (ii) conidial germination into hyphae at 8–12 h; (iii) hyphal development and network expansion at 16–20 h; and (iv) biofilm maturation at 24 h, with formation of an organized mycelial mesh embedded in extracellular matrix (ECM) (Figure 3). The kinetics of these stages depend on inoculum concentration, substrate, temperature, and the strain origin (clinical versus environmental). It should be emphasized that this evidence derives entirely from clinical and soil isolates studied under laboratory conditions; biofilm formation on aquaculture surfaces such as tank walls, RAS biofilters and feed-storage materials has not yet been directly demonstrated and is here inferred from these non-aquaculture models on the basis of the species-level conservation of the trait. The proposed link between *A. fumigatus* biofilm formation and aquaculture infrastructure (RAS pipework, tank walls, feed-storage surfaces) should therefore be regarded as a plausible but still speculative association rather than a confirmed risk mechanism in aquaculture, pending direct demonstration in fish-farming systems.

**Figure 4:**
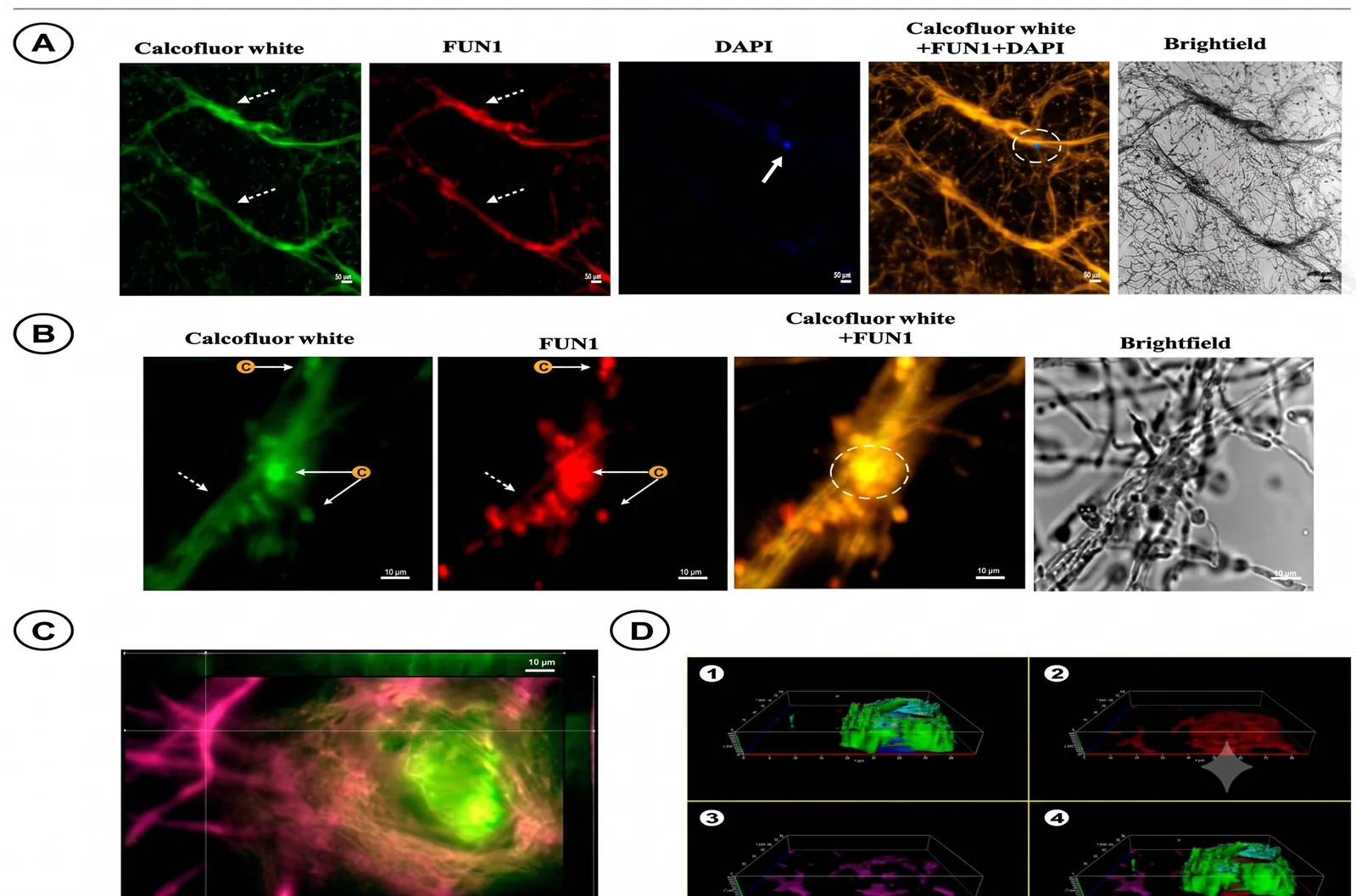
Structural composition of the *Aspergillus fumigatus* biofilm extracellular matrix (ECM) by epifluorescence microscopy (EPM). (**A**) Co-localization of chitin (Calcofluor white, green), metabolic activity (FUN1, red), and nucleic acids (DAPI, blue); yellow halos indicate overlapping components. (**B**) Detection of metabolic activity and chitin, with hyphal anastomosis (white dotted circle). (**C**) Top view of the ECM. (**D**) Three-dimensional z-stack reconstruction of the biofilm showing its molecular components. The ECM (<10 μm thick) embeds the hyphae and protects them from antifungal agents and host defenses. Reproduced from González-Ramírez et al., BMC Microbiology 2016, 16, 243 under CC BY 4.0 license [45].

The mature biofilm is characterized by a complex extracellular matrix composed of polysaccharides (notably galactomannan, galactosaminogalactan, α-1,3-glucans), proteins, chitin, melanin, and extracellular DNA (eDNA). Epifluorescence microscopy with specific fluorochromes allows visualization of these components: Calcofluor white binds chitin (green signal), FUN1 reveals metabolically active cells (red signal), DAPI stains nuclear and extracellular DNA (blue signal), and Flamingo stain marks proteins (magenta signal). Three-dimensional z-stack reconstruction shows that the ECM forms a thin (<10 μm) coherent layer in which hyphae are embedded (Figure 4). The thickness and composition of the ECM are highly relevant to aquaculture because they determine the resistance of environmental biofilms to routine disinfection protocols (chlorine, hydrogen peroxide, quaternary ammoniums) and the persistence of fungal propagules between production cycles.

### 2.3. Affected Fish Species

#### 2.3.1. Freshwater Species

Nile tilapia (*Oreochromis niloticus*) is by far the most frequently reported host of *Aspergillus* infection, reflecting both its global economic importance and its dominant position in the aquaculture industries of Egypt, Southeast Asia, and sub-Saharan Africa [13,14,35,43]. Multiple Egyptian studies describe natural aspergillosis in pond-reared tilapia, with *A. flavus* as the predominant species [12,13]. Mohamed et al. detected *Aspergillus* spp. in 60% of cultured *O. niloticus* and 64% of fish feed in Qena province (Egypt), with *A. flavus* and *A. niger* as the most prevalent species; experimental inoculation with toxicogenic *A. flavus* isolates produced 26.7% mortality within 7 days, and aflatoxin contamination of fish muscles followed the order B1 > B2 > G2 with overall levels below the 20 µg/kg permissible limit [12]. The disease has been reproduced in experimental challenge studies, which have been mainly done in Egypt and Saudi Arabia, enabling the characterization of dose-response relationships, progression of the lesions, and immune responses [37,39,40,46]. In addition to tilapia, aspergillosis has also been reported in silver carp (*Hypophthalmichthys molitrix*), with Iqbal et al. providing a comprehensive morphological and pathological account in the Pakistani culture conditions [8]. African catfish (*Clarias gariepinus*) [35], common carp (*Cyprinus carpio*) [47], and various wild or ornamental freshwater fish have also been reported to be susceptible to natural or experimental infection with *Aspergillus* [7,34].

#### 2.3.2. Marine and Brackish-Water Species

Reports of aspergillosis in farmed marine fish are relatively uncommon, although marine *Aspergillus* strains, in particular those obtained from sponges, sediments and mangroves are becoming increasingly characterized both in terms of their pathogenic potential and their antimicrobial secondary metabolites [27,28,48]. Gilthead sea bream (*Sparus aurata*) and European sea bass (*Dicentrarchus labrax*) are the species that have been examined primarily in the context of dietary aflatoxin exposure, as opposed to direct aspergillosis [49,50], which becomes critical when considering the indirect threat of *Aspergillus* in aquafeeds.

### 2.4. Clinical Signs and Pathogenesis

**Figure 5:**
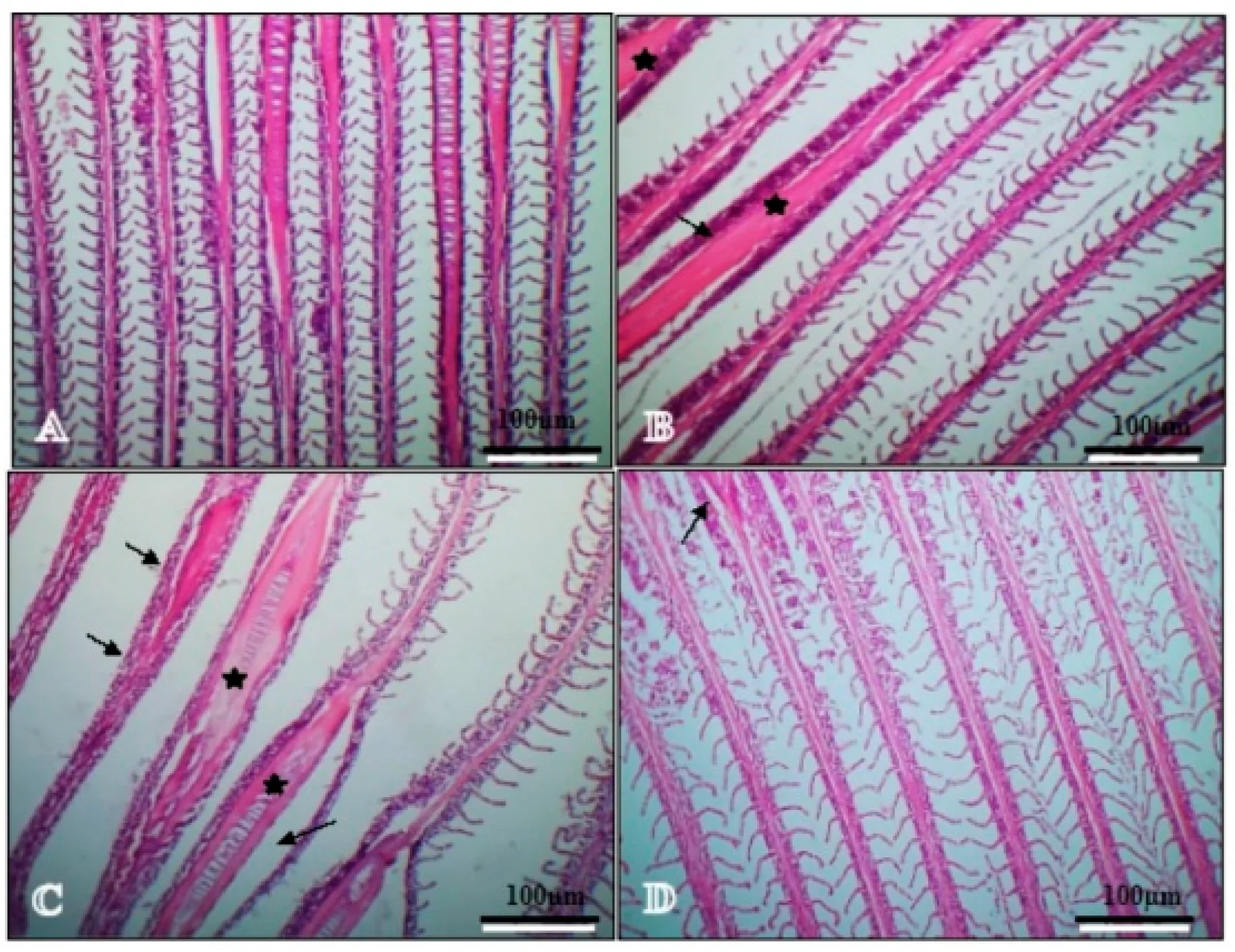
Transverse sections of *Oreochromis niloticus* gill after experimental infection with *Aspergillus flavus*. (**A**) Normal gill architecture (control group); (**B**) normal architecture with some laceration of gill arches (arrows) and hyperplasia and fusion of primary and secondary gill lamellae (stars), nano-Se group; (**C**) vitamin E group showing similar lesions; (**D**) Nano-Se + VE group with light laceration of gill arches only. H&E staining, ×100 magnification, scale bar 100 μm. Reproduced from Bazina et al., BMC Vet Res 2025, 21, 50 under CC BY 4.0 license [40].

In fish, clinical manifestations of aspergillosis are mostly unspecific and can resemble other infectious conditions, including motile aeromonad septicaemia, streptococcosis, or saprolegniasis. Externally, the affected fish usually exhibit lethargy, anorexia, body coloration darkening or paling, loss of scales, erosion of fins, cutaneous haemorrhages, and sometimes opaque corneas [43]. On the inside, the post-mortem examination shows granulomatous lesions in the liver, spleen and anterior kidney extending to the heart, gills, and gastrointestinal tract in a few cases [34] (Figure 5). Iqbal et al. reported clinical manifestations such as ruptured skin, eroded scales, damaged fins, and cataract in eyes in silver carp that were naturally infected with *Aspergillus*, which is in line with mycotic infection [8].

Understanding aspergillosis in fish needs to be perceived as an opportunistic disease. *Aspergillus* conidia are common in the pond and tank settings but the clinical disease only occurs when host defenses are weakened or when the pressure of the fungus is beyond the immunological tolerance. The most commonly reported predisposing factors include high stocking density, low water quality (low dissolved oxygen, ammonia or nitrite buildup), nutritional imbalances, co-occurring bacterial or parasitic infections, and chronic exposure to moldy feed [26,34,51]. Such opportunistic nature is an important concept since it is precisely this that enables the same genus, under different conditions, to act as pathogens, commensals or even probiotics.

### 2.5. Histopathology

Histopathology is a pillar of fish aspergillosis diagnostics and, in the experimental research, one of the most informative outcomes of determining the extent of infection and the effectiveness of dietary intervention. Lesions are typical but not pathognomonic, and involve the gills, intestine, and hepatopancreas, which are the three most frequently studied organs in aquaculture-oriented research. In the gills, *A. flavus* challenge produces laceration of gill arches, hyperplasia and fusion of primary and secondary lamellae, and, in severe cases, branchial remodelling with compromised respiratory function (Figure 5). Bazina et al. reported that nano-selenium (SeNPs) and vitamin E (VE), especially in combination, markedly attenuated these branchial lesions in Nile tilapia [40], confirming earlier findings with nano-curcumin [37].

**Figure 6:**
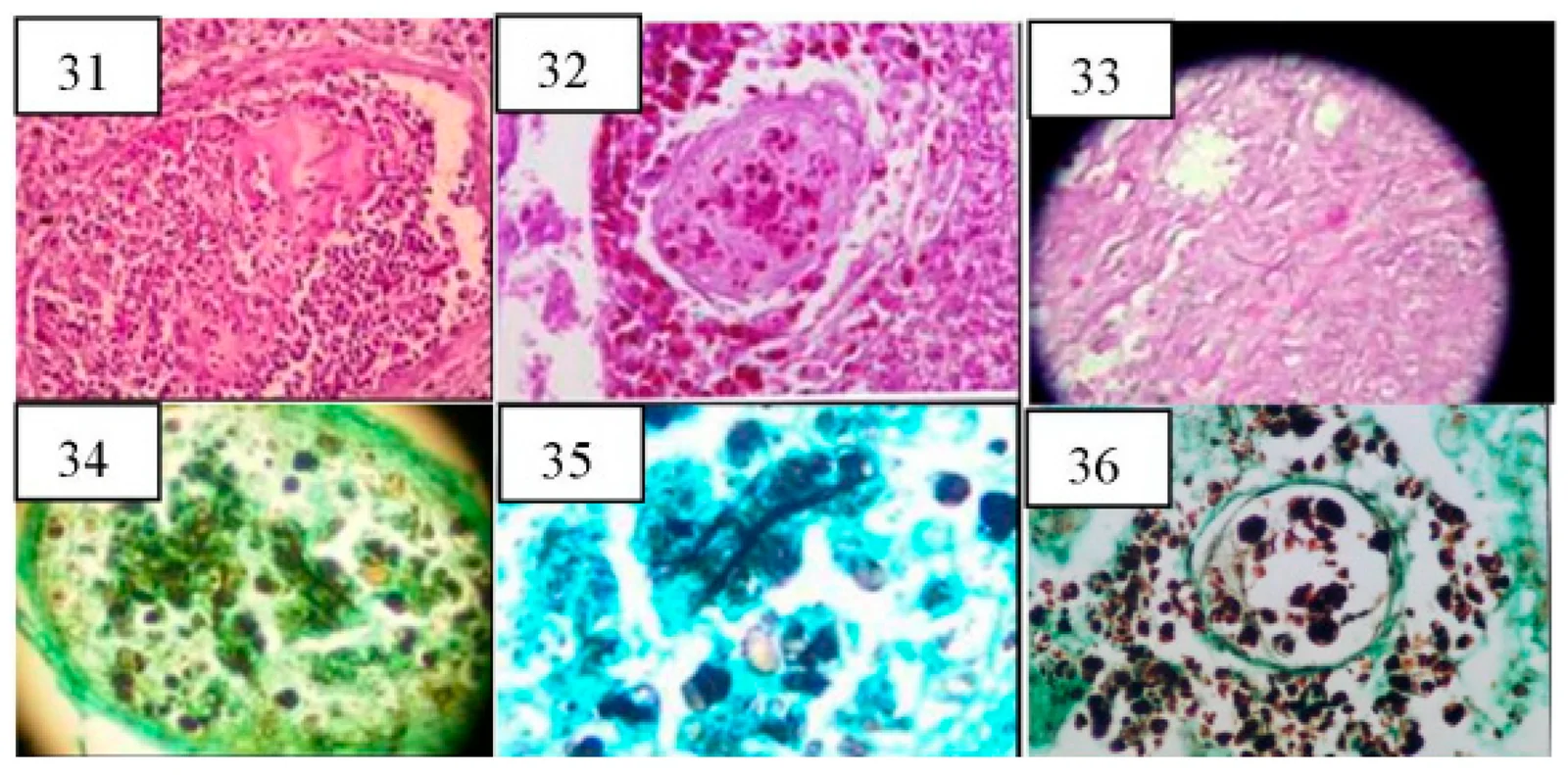
Histopathology of fungal granulomas in the spleen and liver of cultured freshwater fish (*Oreochromis* spp. and *Clarias gariepinus*) naturally infected with *Aspergillus* species in Egypt. (31) Spleen section (PAS, ×400) showing a granuloma formed of epithelioid cells and macrophages surrounded by fibroblasts and a fibrous connective tissue capsule, with fungal hyphae visible within the granuloma; (32) Spleen section (PAS, ×400) showing a granuloma with epithelioid cells, macrophages and connective tissue capsule, with numerous fungal spores within and surrounding the granuloma; (33) Liver section (PAS, ×200) showing fungal hyphae between the hepatocytes; (34) Liver section (GMS, ×400) showing a granuloma comprising aggregated epithelioid cells, macrophages and fibrous connective tissue capsule, with fungal hyphae and spores within the granuloma; (35) Liver section (GMS, ×1000) showing fungal hyphae and spores between hepatic cells; (36) Spleen section (GMS, ×400) showing focal aggregation of spores surrounded by proliferating fibroblasts and fibrous connective tissue. Reproduced with permission from Refai et al., Journal of American Science 2010, 6(11): 823–831 [34]. Photographs by Shimaa E. Ali.

Naturally infected fish from Egyptian aquaculture facilities exhibit characteristic granulomatous lesions in the spleen and liver, with epithelioid cells, macrophages and fungal hyphae visible by PAS and GMS staining (Figure 6) [34].

**Figure 7:**
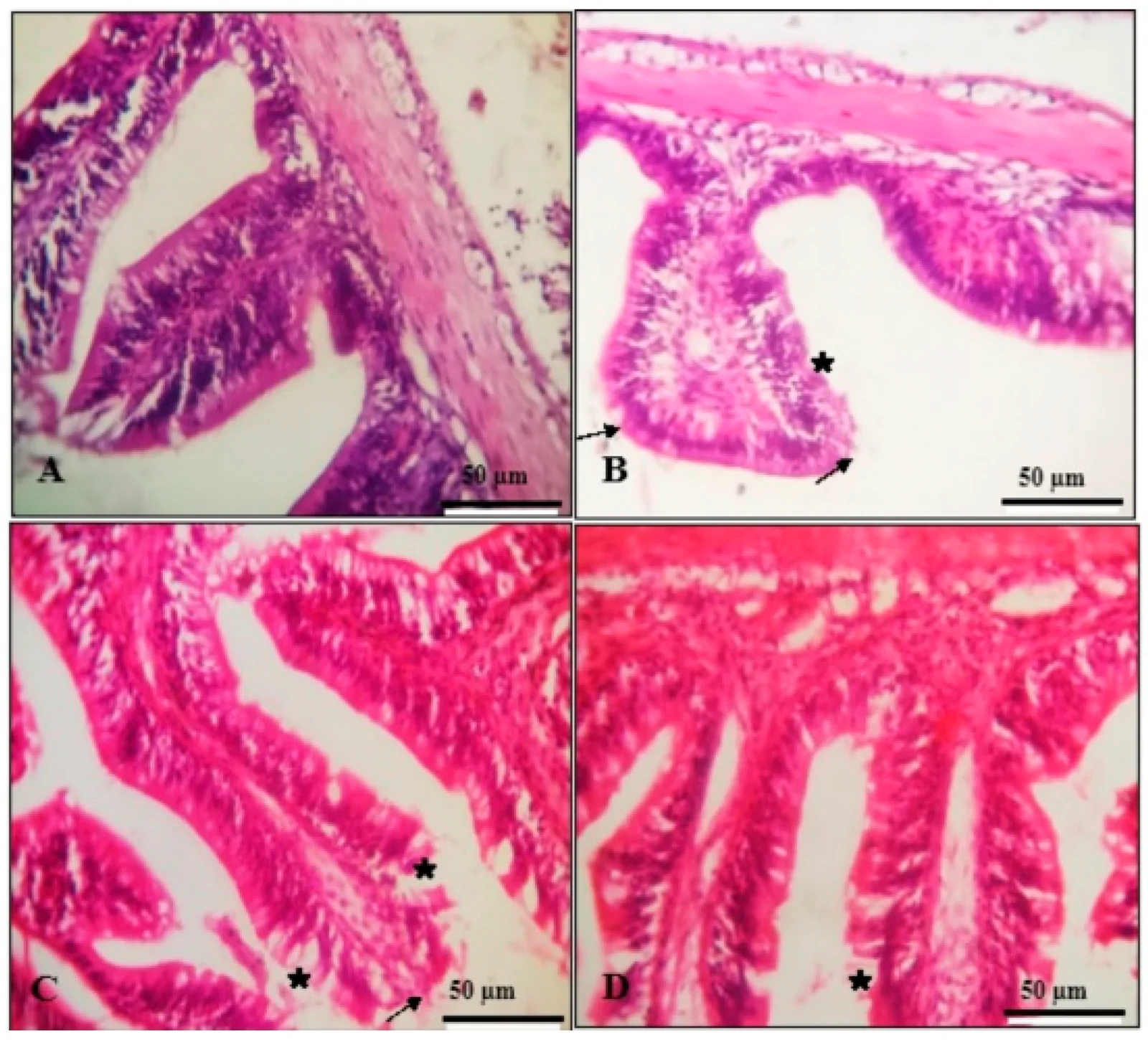
Transverse sections of *Oreochromis niloticus* intestine after *Aspergillus flavus* challenge. (**A**) Normal structure (control); (**B**) light deformation in villi cell structure (arrows) with degeneration of surface epithelial cells (stars), nano-Se group; (**C**) vitamin E group showing similar but reduced lesions; (**D**) Nano-Se + VE group showing normal structure with marked improvement and elongation of villi length. H&E staining, ×400 magnification, scale bar 50 μm. Reproduced from Bazina et al., BMC Vet Res 2025, 21, 50 under CC BY 4.0 license [40].

In the intestine, *A. flavus* infection disrupts the structure of the mucosal villi: villi become deformed, epithelial enterocytes degenerate, and the absorptive surface is reduced, changes that collectively compromise nutrient uptake and contribute to the growth depression repeatedly documented in aspergillosis-affected fish. Bazina et al.’s H&E sections at ×400 magnification illustrate this pattern and demonstrate the protective effect of dietary antioxidants: in the SeNPs + VE group, intestinal architecture was largely preserved with elongated villi, consistent with improved digestive function (Figure 7) [40]. Enteric lesions of a similar nature have been reported in tilapia fed aflatoxin-contaminated diets and in experimentally challenged fish [46,52,53].

**Figure 8:**
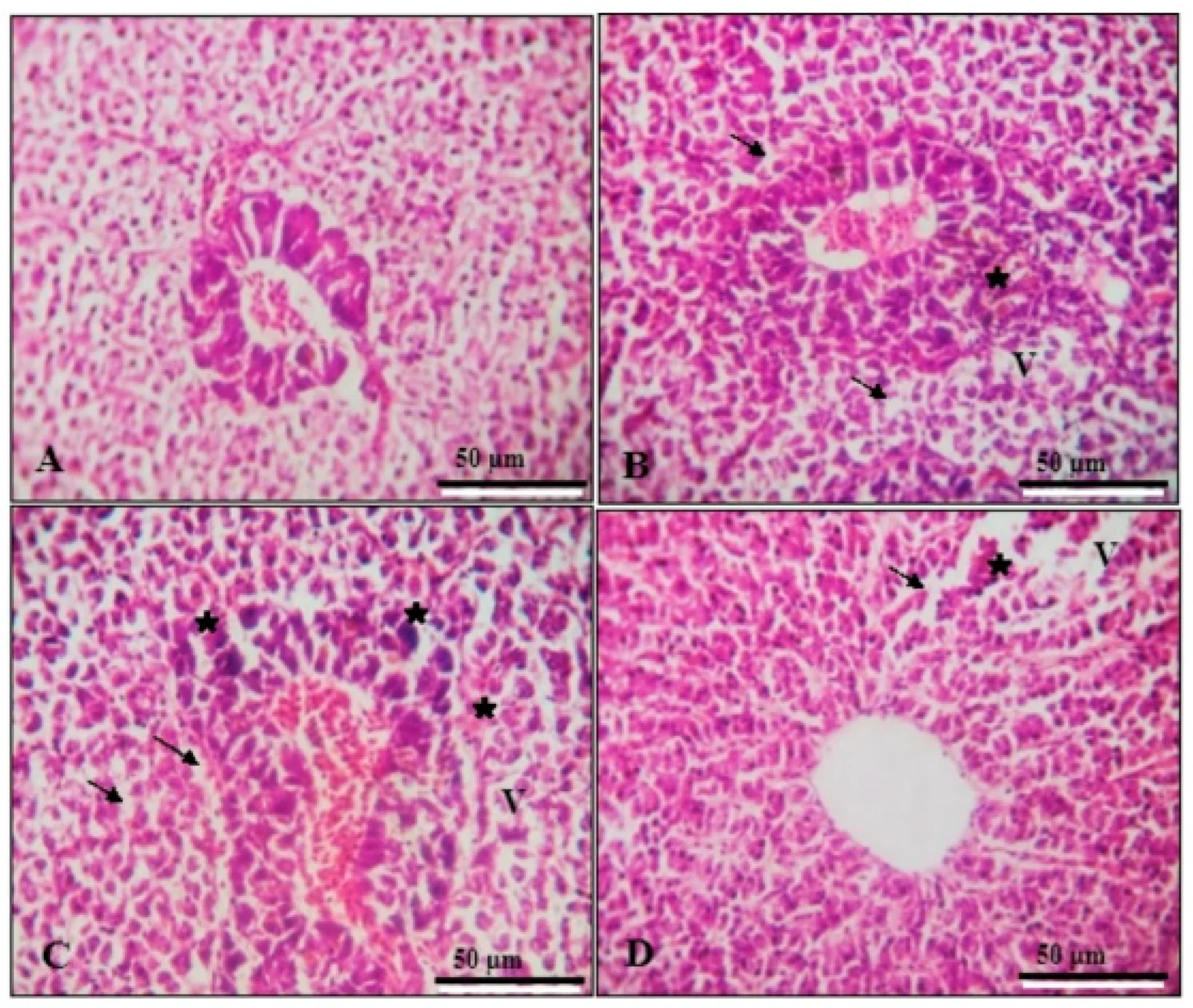
Transverse sections of *Oreochromis niloticus* hepatopancreas after *Aspergillus flavus* challenge. (**A**) Normal structure of hepatic cords, pancreatic acini and vascular tissue (control); (**B**) normal morphology of portal veins and hepatic sinusoids with some vacuolated hepatocytes (V), hepatocyte degeneration (arrows), mild pancreatic islands congestion (stars), nano-Se group; (**C**) vitamin E group; (**D**) Nano-Se + VE group with restored normal architecture. H&E staining, ×400 magnification, scale bar 50 μm. Reproduced from Bazina et al., BMC Vet Res 2025, 21, 50 under CC BY 4.0 license [40].

The liver (hepatopancreas) is the organ most severely and consistently affected in fish aspergillosis, reflecting both its central role in fungal metabolite detoxification and its anatomical proximity to the portal venous drainage of the gut. Classical lesions include vacuolar degeneration of hepatocytes, hepatocyte necrosis, congestion of pancreatic islands, and disruption of the normal architecture of hepatic cords [37,39,40]. Periodic acid-Schiff (PAS) and Grocott’s methenamine silver (GMS) stains reveal septate, dichotomously branching hyphae, approximately 3–6 µm in diameter, radiating from necrotic cores into surrounding inflammatory infiltrates. Figure 8 illustrates this pathology and the restorative effect of combined nano-Se + VE supplementation, under which hepatopancreatic architecture is largely preserved. Splenic and renal lesions follow a similar granulomatous pattern, often accompanied by melanomacrophage center proliferation, a classic indicator of chronic antigenic stimulation in fish [34,46].

**Figure 9:**
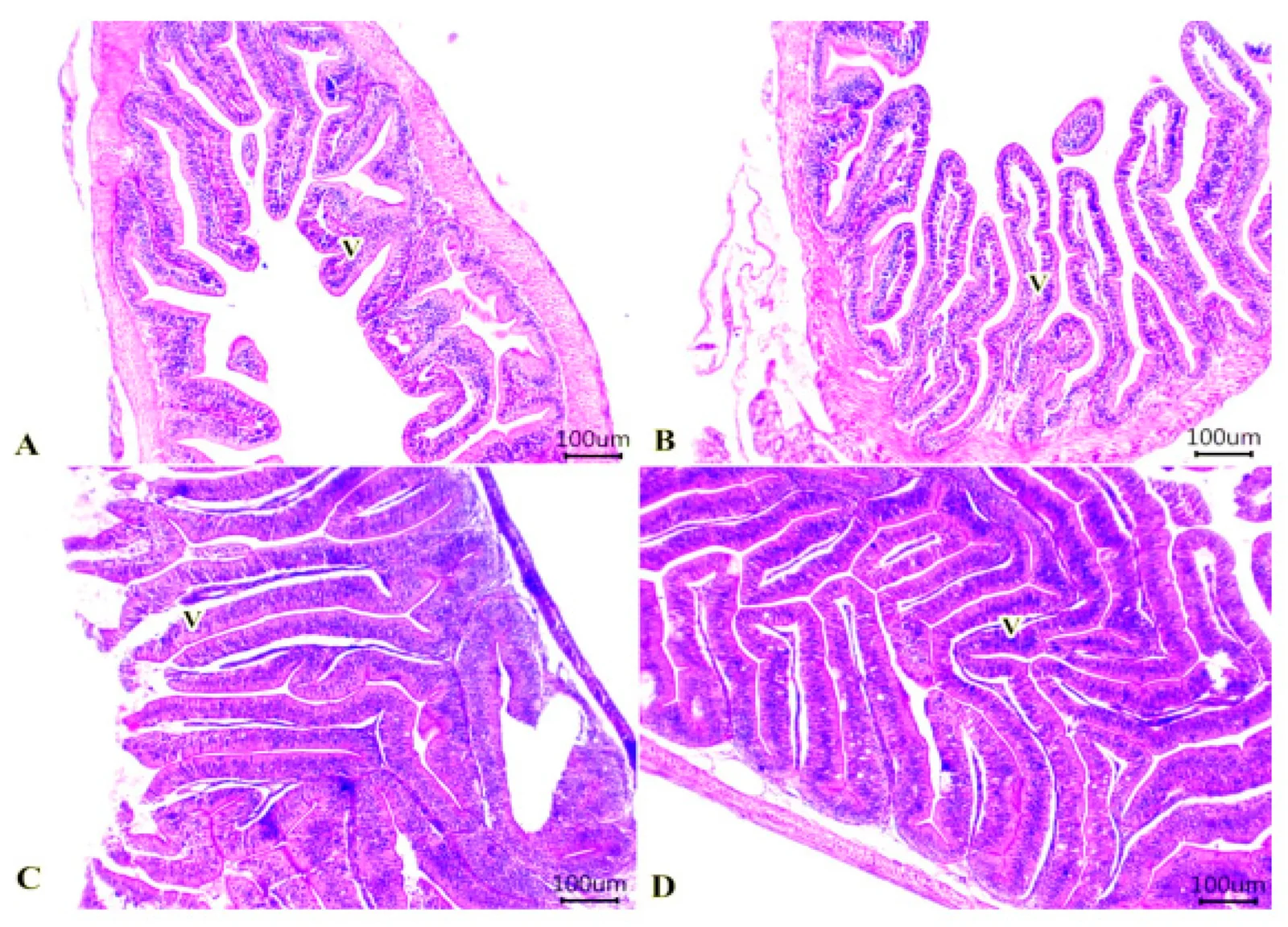
(**A**–**D**) Photomicrographs of H&E-stained sections from the intestine of *Oreochromis niloticus* fed *Phyllanthus emblica* at increasing dietary levels. (**A**) Normal structures of mucosal villi (V), submucosa, and muscularis in the control group; (**B**–**D**) increased number of enterocytes lining the villi in intestinal fish fed diets with 1% (T1), 2% (T2) and 3% (T3) of *P. emblica* powder for 60 days, respectively. Scale bar 100 μm. Reproduced from Jastaniah & Albaqami, Scientific Reports 2025, 15, 11226 under CC BY 4.0 license [38].

The histopathological protection afforded by dietary interventions extends beyond nano-minerals and vitamins to include plant-derived immunostimulants. Jastaniah and Albaqami reported that dietary supplementation of Nile tilapia with *Phyllanthus emblica* (Indian gooseberry) powder at 1%, 2%, and 3% for 60 days improved intestinal and hepatic histoarchitecture in a dose-dependent manner [38]. At the intestinal level, *P. emblica*-fed fish displayed a progressively higher number of enterocytes lining the villi, compatible with enhanced digestive and absorptive function (Figure 9). The protective effect of *P. emblica* is attributable to its rich phytochemical profile, including gallic acid, resveratrol, quercetin, lignans, cyanidin, genistein, and hesperitin, which collectively confer antioxidant, anti-inflammatory and immunomodulatory properties relevant to fish challenged with *A. flavus* [38]. These findings add to the growing evidence that plant-derived polyphenols and antioxidants can mitigate the structural damage caused by *A. flavus* in cultured fish [37,43].

**Figure 10:**
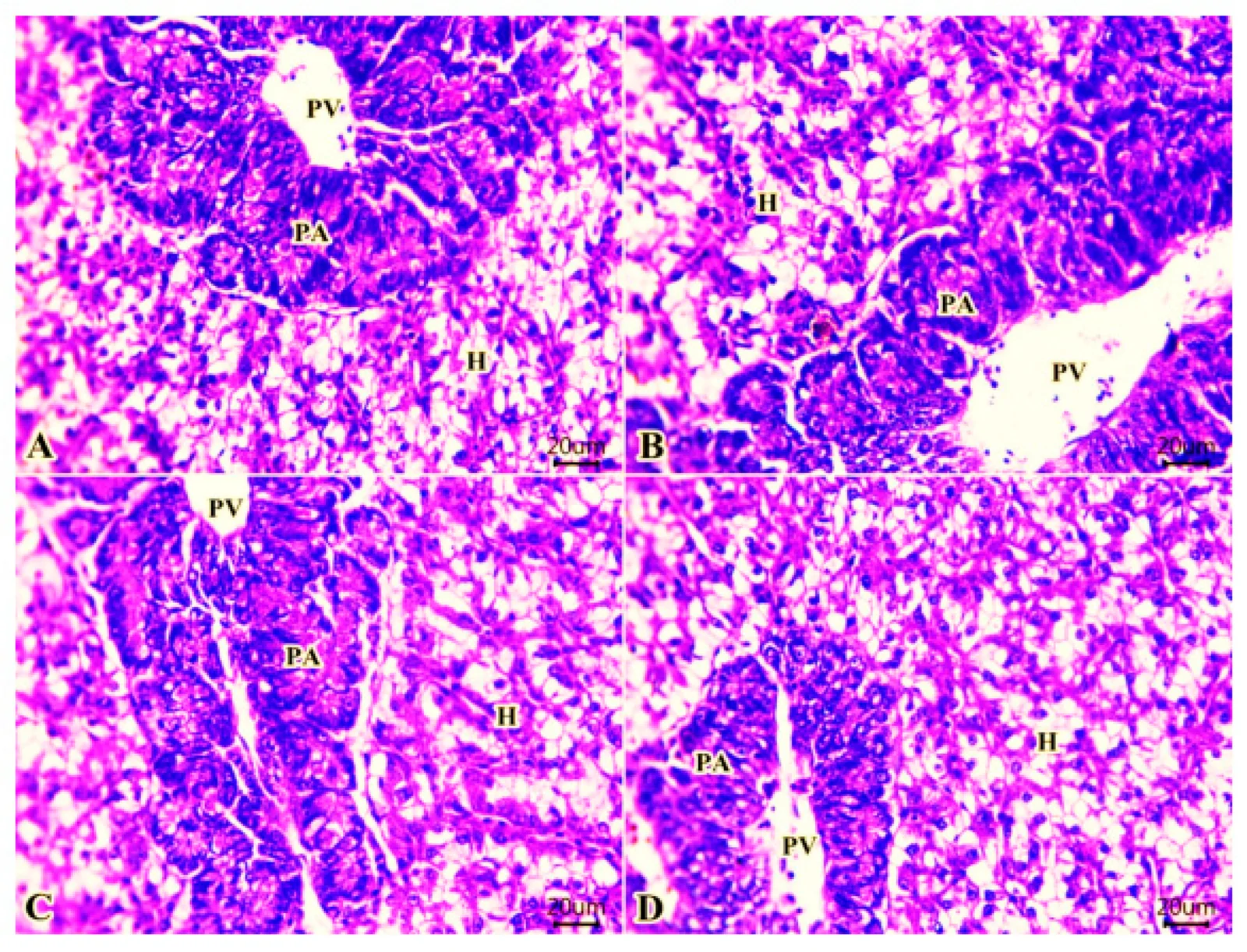
(**A**–**D**) Photomicrographs of H&E stained liver sections of *Oreochromis niloticus*. (**A**) Normal morphology of hepatic cords with vacuolated areas alongside normal hepatopancreatic acini and portal vein in the control group; (**B**–**D**) hepatic tissues of fish displaying preserved hepatocytes, hepatoportal pancreas, portal veins, central veins, and sinusoids in groups T1 (1%), T2 (2%) and T3 (3%) *P. emblica*, respectively. Annotations: Hepatocytes (H), Pancreatic acini (PA), Portal vein (PV). Scale bar 20 μm. Reproduced from Jastaniah & Albaqami, Scientific Reports 2025, 15, 11226 under CC BY 4.0 license [38].

At the hepatic level, the same study documented preservation of hepatocyte cords, hepatopancreatic acini, portal veins, and sinusoids uniformly across all three *P. emblica* inclusion levels (T1, T2, T3), with hepatocytes (H), pancreatic acini (PA), and portal veins (PV) maintaining normal morphology compared to the control group (Figure 10) [38].

Taken together, these histopathological studies in Nile tilapia consistently demonstrate that *A. flavus* challenge induces measurable structural damage in gills, intestine, and hepatopancreas, and that dietary antioxidant or phytogenic supplementation can substantially mitigate these lesions. From a practical standpoint, histopathological scoring therefore constitutes a valuable and quantifiable endpoint in aquaculture-oriented experimental studies [37,38,39,40,46].

### 2.6. Diagnostic Approaches

Traditional diagnosis of fish aspergillosis involves clinical examination, gross post-mortem lesions, direct microscopic examination (wet mounts, tissue imprints), and fungal culture on Sabouraud dextrose agar (SDA) or potato dextrose agar (PDA) with chloramphenicol [14,34,35]. Colony appearance, conidial head shape and microscopic characteristics (arrangement of phialides, conidial ornamentation and colour) can provide provisional species identification. But morphology is not enough to differentiate between toxigenic and non-toxigenic strains, and species in *Aspergillus* sections Flavi and Nigri are not only morphologically similar but also difficult to distinguish without molecular tools. Molecular techniques, including PCR amplification and sequencing of the internal transcribed spacer (ITS) sequences and *calmodulin* or β-tubulin genes, are now routinely used in well-resourced labs [13,35]. Abd El Tawab et al. showed that molecular diagnosis of *Aspergillus* isolates from cultured Nile tilapia in Egypt by PCR amplification of the ITS gene identified both *A. flavus* and *A. niger* [13]. However, molecular analyses are not always available to farmers, and many fish aspergillosis cases are still only diagnosed to the genus level. Accordingly, a diagnosis of fish aspergillosis should be qualified by its level of diagnostic certainty: a suspected case, based on clinical signs and gross post-mortem lesions; a culture-based case, based on fungal growth and provisional morphological identification on SDA/PDA; a histopathologically confirmed case, based on demonstration of septate, dichotomously branching hyphae within granulomatous lesions by PAS or GMS staining; and a definitively identified case, based on molecular confirmation by ITS, β-tubulin or calmodulin sequencing.

### 2.7. Predisposing Factors

The opportunistic nature of fish aspergillosis makes management rather than treatment a key strategy. Three types of predisposing factors have been consistently noted. The first is environmental: warm water, poor water quality, organic loading and, in open production, the presence of high spore counts in pond sediments and soils [26,34]. The second is host-specific: immunosuppression associated with stress, nutritional deficiencies (especially vitamins E and selenium and omega-3 fatty acids), overstocking, co-infections and improper handling [54]. The third is feed-related: the ingestion of moldy feed components not only provides a direct fungal challenge but also introduces aflatoxins that affect the immunity of the fish, resulting in a vicious cycle between feed fungal contamination and fish mycosis [25,26].

### 2.8. Treatment and Prevention

Therapeutic options for fish aspergillosis are severely limited. Most antifungal drugs approved in human or small-animal medicine (amphotericin B, azoles, echinocandins) are not registered for food fish, and their use raises concerns about residues, cost, and environmental release [55]. As a consequence, control strategies have focused on prevention and on adjunctive, non-pharmacological interventions. Several lines of evidence have emerged.

First, dietary supplementation with antioxidant nanomaterials has shown promise. Bazina et al. demonstrated that nano-selenium alone or in combination with vitamin E improved survival, growth, antioxidant status, and histopathological scores in Nile tilapia experimentally challenged with *A. flavus* [39,40]. Eissa et al. reported similar protective effects of nano-curcumin in red tilapia [37], and Jastaniah and Albaqami documented beneficial effects of *Phyllanthus emblica* (Indian gooseberry) extract against *A. flavus* infection in Nile tilapia [38]. El-Zayat et al., working in Aswan Governorate, Egypt, showed that the aquatic plant *Persicaria salicifolia* can act as a natural antifungal against *A. flavus* in tilapia, offering a low-cost, locally available intervention for small-scale farmers [43].

Second, bacterial probiotics have been repeatedly shown to enhance fish resistance to subsequent *A. flavus* challenge. Dighiesh et al. demonstrated that multi-strain *Bacillus* supplementation improved growth, blood biochemistry, intestinal histology, and disease resistance in Nile tilapia challenged with *A. flavus* [46]. Two separate reports by Eissa et al. and Hendam et al. demonstrated protective effects of the probiotic *Pediococcus acidilactici*, either as a feed supplement or water additive, against experimentally infected Nile tilapia with *A. flavus* [52,53]. More recently, Shokrak et al. reported *Bacillus rugosus* NM007 as a new probiotic strain, which increased growth, immune response and intestinal histomorphology in Nile tilapia and showed that the flow of potential probiotic strains for aquaculture is not exhausted yet [56]. These findings demonstrate an ecological principle: biological control of pathogens via probiotic competition and the production of natural antifungal metabolites can be applied in aquaculture, either as an addition to or replacement for pharmaceutical antifungals.

Third, biosecurity and management are key to prevention. Feeding high-quality formulated feed, maintaining low humidity (less than 12%), exclusion of rodents and insects, short storage times, regular water quality monitoring, quarantine of new stock, and disinfection of nets, tanks and hatchery equipment are essential [51]. The spread and emergence of multidrug-resistant pathogens in tilapia aquaculture, such as *Streptococcus agalactiae* in India [57] and pathogenic *Edwardsiella anguillarum*, *Nocardia asteroides* and *S. agalactiae* in Mexican tilapia farms [58], also highlight the need for integrated disease control measures, including fungal pathogens. In addition to mycotic diseases, vaccination strategies are also rapidly evolving in tilapia aquaculture, as shown in Padhiary et al.’s recent assessment of systemic and mucosal immune responses to inactivated tilapia lake virus vaccines [59], which reinforces the idea that integrated immune-based strategies are now at the forefront of fish health. An integrated management approach that includes good management, feed quality control, and specific dietary additives appears to be, at the moment, the only viable solution to control aspergillosis in fish farms.

### 2.9. Economic Impact

The global economic burden of fish aspergillosis is unknown, consistent with under-reporting mentioned above. However, local outbreaks can lead to cumulative mortality of 20–60% in infected ponds, with significant indirect losses from reduced growth, lower market value and treatment costs [39]. In tilapia-consuming countries, recurrent fungal infections affect producer livelihoods and food security; recent stochastic frontier analyses of Egyptian tilapia farms have estimated an average technical efficiency of 0.79 and identified the number of species cultured, age of the farming facilities, and farmer education as significant predictors of farm efficiency and sustainability in the long term [2]. The likely dependence of *Aspergillus* growth on climatic factors suggests that the economic impact of aspergillosis will increase under climate change scenarios that project higher water temperatures, extreme weather and feed storage issues in tropical countries [21,26]. Table 2 summarizes the hosts, clinical and pathological signs, diagnostic methods, and preventive strategies of pathogenic *Aspergillus* species.

**Table 2:** Pathogenic *Aspergillus* species in finfish: hosts, clinical and pathological signs, diagnostic methods, and preventive strategies.

*Aspergillus* Species	Main Fish Host(s)	Key Clinical & Pathological Signs	Diagnostic Methods	Preventive/Control Strategies	Ref.
*A. flavus*	Nile/red tilapia, common carp, silver carp	Lethargy, anorexia, skin darkening/paling, scale loss, fin erosion, cutaneous haemorrhage, opaque cornea; granulomatous lesions of liver, spleen and kidney; branchial, intestinal and hepatopancreatic damage	Gross lesions; culture (SDA/PDA); histopathology (PAS/GMS); PCR–ITS sequencing	Feed quality control; dietary antioxidants (nano-Se, vitamin E, nano-curcumin); plant immunostimulants; probiotics; biosecurity	[8,12,13,37,38,39,40,43,46]
*A. niger*	Common carp, African catfish, freshwater fish	Mycotic lesions; granulomatous inflammation; systemic involvement in experimental challenge	Culture; microscopy; PCR–ITS	Feed and storage hygiene; biosecurity; probiotic competition	[7,13,35,41]
*A. fumigatus*	Tilapia (dominant isolate, Indonesia); freshwater fish	Severe systemic disease; environmental persistence via biofilm on tank and RAS surfaces	Culture; microscopy; molecular ID (ITS/β-tubulin/calmodulin)	Disinfection; humidity and biofilm control; biosecurity	[7,14,45]
*A. terreus*, *A. sydowii*	Freshwater fishes (Channa, Clarias)	Less commonly reported; severe systemic disease in experimentally challenged fish	Culture; molecular identification	Biosecurity; water-quality management	[7]

## 3. *Aspergillus* as an Indirect Threat: Aflatoxins in Aquaculture

### 3.1. Aflatoxins: Chemistry and Toxigenic Species

Aflatoxins are a family of polyketide-derived secondary metabolites produced principally by *A. flavus* and *A. parasiticus* [22]. The four naturally occurring aflatoxins, B1, B2, G1, and G2, differ in the presence or absence of a cyclopentenone ring and in the nature of the bisfuran moiety that governs their genotoxic potential.

**Figure 11:**
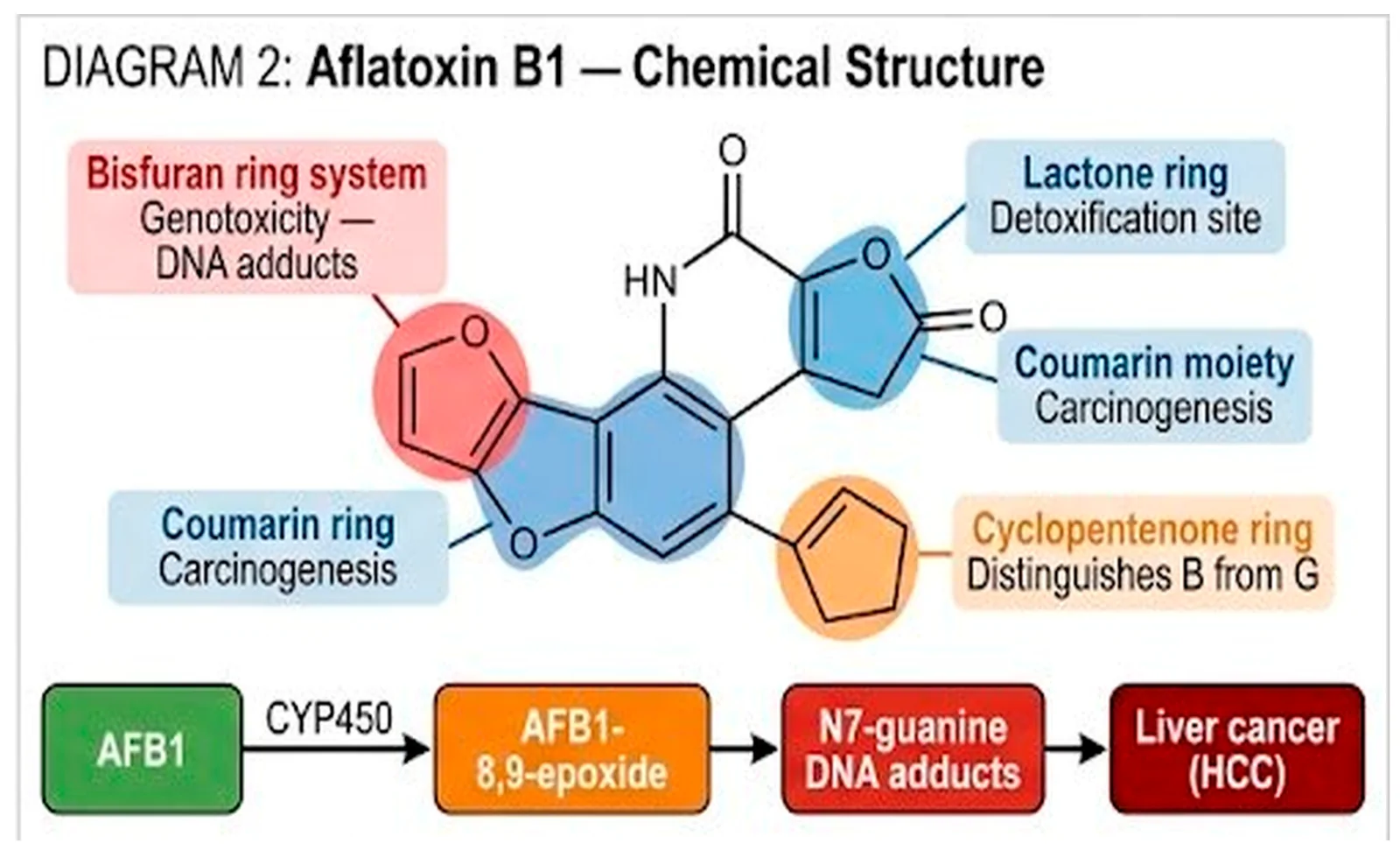
Chemical structure of aflatoxin B1 (AFB1, C_17_H_12_O_6_, MW 312.27 g/mol), a Group 1 carcinogen (IARC). Key reactive groups are highlighted: the bisfuran moiety responsible for genotoxicity (DNA adducts), the lactone ring (target of detoxification), the coumarin moiety (carcinogenicity), and the cyclopentenone ring distinguishing B from G aflatoxins. The lower panel illustrates the bioactivation pathway in fish: AFB1 is metabolized by hepatic CYP450 enzymes to the highly reactive AFB1-8,9-epoxide, which binds DNA and proteins forming N7-guanine adducts that ultimately lead to hepatocellular carcinoma.

Aflatoxin B1 (AFB1) is the most significant in toxicological and regulatory aspects: it is a Group 1 carcinogen to humans, the most potent naturally occurring hepatotoxin, and the main aflatoxin found in aquaculture feeds globally [22,25,26]. AFB1 structure and the pathway for its bioactivation in the fish liver are depicted in Figure 11. The molecule is divided into four functional parts: a bisfuran ring that causes genotoxicity through DNA adduct formation, a lactone ring that is the main site for enzymatic and/or chemical detoxification, a coumarin ring that forms the backbone of carcinogenicity and a cyclopentenone ring that is used to differentiate between B-type (B1, B2) and G-type (G1, G2) aflatoxins. When ingested with contaminated feed, AFB1 is absorbed and transported to the liver where it is enzymatically converted to AFB1-8,9-epoxide by cytochrome P450 (CYP450) enzymes. The electrophile intermediate covalently binds to DNA, RNA, and proteins, inactivates the p53 tumor suppressor gene by GC→TA mutations, and finally triggers hepatic carcinogenesis. AFB1-DNA adducts have been identified in the liver of AFB1-treated rainbow trout and Atlantic salmon, and the prolonged half-life of these adducts in fish hepatocytes compared to mammals suggests an increased risk of mutation in fish [60]. In milk-producing mammals, AFB1 is partially hydroxylated to aflatoxin M1 (AFM1), which can be found in milk and, presumably, in aquatic food products in cases of chronic exposure in fish [25].

### 3.2. Contamination of Aquafeeds: Global Picture

**Figure 12:**
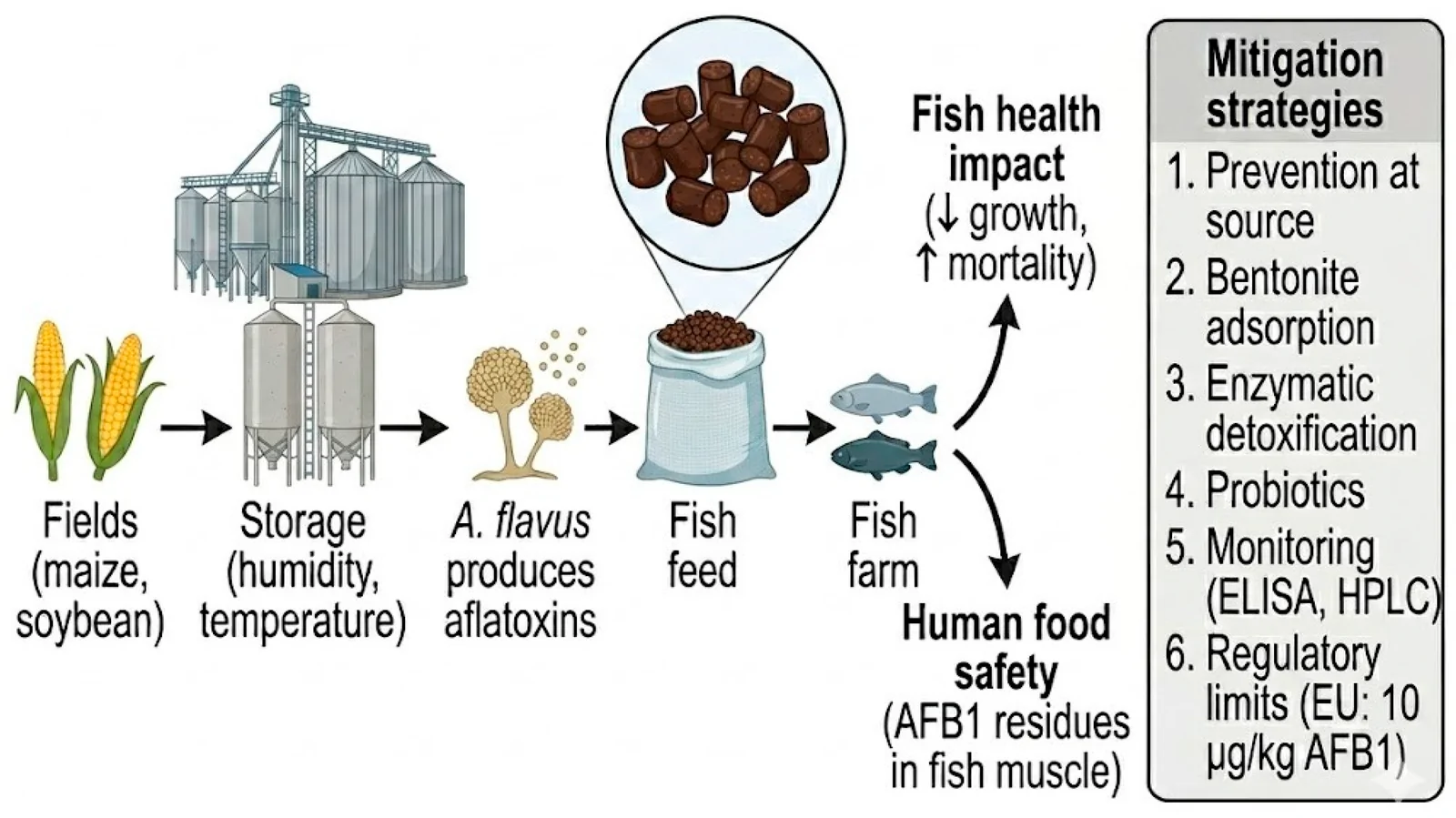
Aflatoxin contamination in the aquaculture feed chain. Agricultural crops (corn, soy, peanut, cottonseed) inoculated by *A. flavus*/*A. parasiticus* under drought conditions are stored in conditions conducive for fungal growth (moisture, temperature, insects) where aflatoxin is produced through the aflR/aflS-regulated gene cluster. The mycotoxins are transferred through aquafeed production to fish via chronic feeding, and cause two linked consequences: (i) fish health problems (growth, immune function, survival; mortality, tissue damage) and (ii) food safety issues (AFB1/AFM1 residues in fish muscle consumed by humans). Six categories of mitigation strategies are summarized on the right. Notably, the European Union regulatory limit of 10 µg/kg AFB1 for complete and complementary animal feeds under Directive 2002/32/EC applies to fish within the general ‘other animals’ category, as no aquaculture-specific regulatory threshold has been established to date, a regulatory gap recently highlighted in the literature [21].

Aflatoxin contamination of aquafeeds begins well before the feed reaches the fish farm. The full contamination cascade, from pre-harvest fungal infection of field crops through storage, manufacturing, and ultimate delivery to farmed fish, is summarized in Figure 12. Field crops such as maize, soybean, peanut, and cottonseed become increasingly susceptible to *A. flavus* and *A. parasiticus* colonization under drought and heat stress, two conditions that are expected to intensify under climate change. Post-harvest storage under high humidity, elevated temperature, and insect infestation creates the conditions in which the fungi colonize the grain matrix and switch on the aflatoxin biosynthetic pathway [22]. Toxigenic production requires permissive substrate and environmental conditions: temperatures above 27 °C, humidity levels above 62%, feed moisture greater than 14%, damaged or cracked grain surfaces, and extended storage [25]. AFB1, once produced, is chemically stable and survives the heat and pressure of aquafeed pelletization, so it reaches fish farms in the finished feed [26]. Chronic ingestion then produces two interconnected outcomes: (i) direct fish health impacts (growth depression, immunosuppression, organ damage, mortality); and (ii) food safety concerns linked to the carry-over of AFB1 and AFM1 residues into edible fish tissues, reaching the human consumer and constituting a One Health issue that links aquafeed quality to public health. Mycotoxin mixtures pose additional concerns: AFB1 and zearalenone can exert synergistic toxicity in salmonids such as rainbow trout, though this effect may be partially attenuated by anabolic dietary interventions [61].

The replacement of fishmeal by plant-based protein and carbohydrate ingredients, mostly soybean meal, maize, cottonseed meal, peanut meal, rice bran, and wheat by-products, has mechanically increased the exposure of aquafeeds to mycotoxigenic fungi, because these ingredients are themselves frequently contaminated at source [23,25,26,62]. The most comprehensive recent global survey, conducted by Gruber-Dorninger et al. on 226 fish feed and 61 shrimp feed samples collected worldwide, documented that more than 90% of compound feeds contained at least one mycotoxin, that 7.2% of fish feed samples exceeded the European Union maximum level of 10 µg/kg for AFB1, and that co-contamination with multiple mycotoxins was the rule rather than the exception [24]. This 10 µg/kg threshold, established by Directive 2002/32/EC for complete and complementary animal feeds, remains the sole European regulatory limit currently in force for AFB1 in aquafeeds [21]. Critically, this threshold is not aquaculture-specific but is adapted from general animal-feed legislation, and regional aflatoxin limits are likewise defined for animal feed as a whole rather than for aquafeed, so that no jurisdiction has yet established a dedicated maximum level for AFB1 in fish feed. A further complication, highlighted in the recent review by Bittner et al., concerns the accelerating use of insect-based ingredients as alternative protein sources: insects themselves can be exposed to mycotoxins through their rearing substrates, potentially introducing additional contamination pathways into aquafeed supply chains [22]. Surveys across continents have repeatedly identified *Aspergillus* spp. and aflatoxins in aquafeeds. Mwihia et al. documented the occurrence and levels of aflatoxins in fish feeds from Nyeri, Kenya, and highlighted the risk for tilapia farmers relying on locally formulated feeds [63]. Barbosa et al. characterized the mycobiota and mycotoxin profiles of finished fish feeds from farms in Rio de Janeiro State, Brazil, and reported AFB1 in 55% of samples, with co-occurrence of fumonisin B1 and ochratoxin A [64]. Greco et al. analyzed rainbow trout (*Oncorhynchus mykiss*) feeds and identified a natural co-occurrence of aflatoxins and *Fusarium* toxins, underlining the multi-contaminant nature of the problem [65]. Table 3 summarizes representative regional data.

### 3.3. Toxic Effects on Fish

Fish species differ in their sensitivities to AFB1. Rainbow trout (*Oncorhynchus mykiss*) are classically regarded as very sensitive, with liver tumor development after chronic exposure to low parts per billion (ppb) concentrations [65]. Gilthead sea bream (*Sparus aurata*) exhibit dose-dependent growth depression, altered function of the somatotropic axis, metabolic alterations and histopathological lesions in the liver, intestine and kidney after dietary exposure to AFB1 [49,66]. European sea bass (*Dicentrarchus labrax*) reared in marine water exhibit biochemical alterations and measurable AFB1 residues in edible tissues when exposed to contaminated diets [50]. Nile tilapia, generally considered more resistant than cold-water salmonids, nevertheless shows growth depression, oxidative stress, hepatic and renal lesions, immunosuppression, and measurable AFB1 bioaccumulation when fed contaminated diets [67,68]. Across species, the main toxic endpoints are hepatotoxicity and hepatocarcinogenesis, immunosuppression, oxidative stress, endocrine disruption, reduced growth, and, in severe cases, mortality [22,25,26].

**Figure 13:**
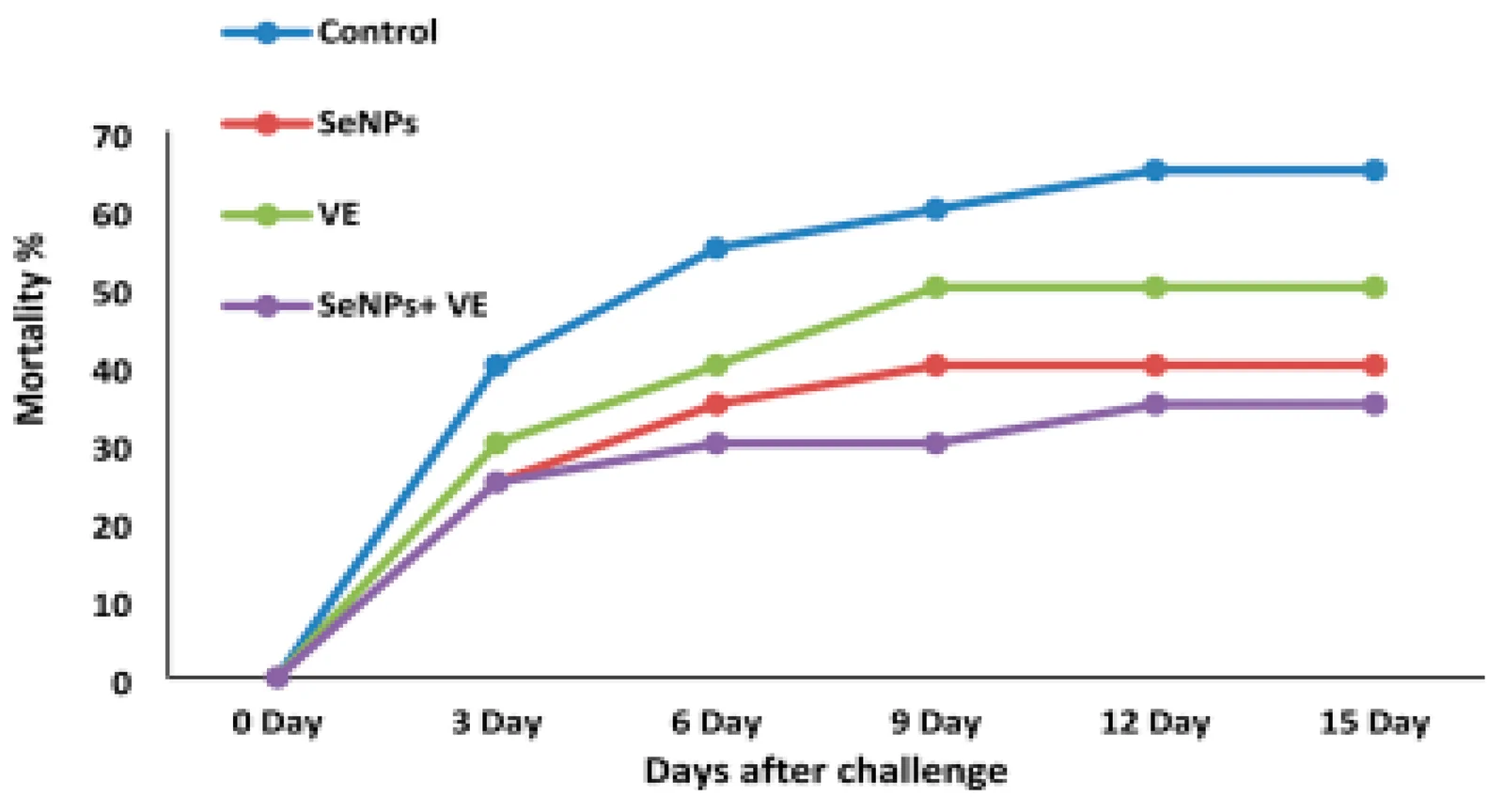
Cumulative mortality of *Oreochromis niloticus* after experimental challenge with *Aspergillus flavus* over 15 days, showing the protective effects of dietary antioxidant supplementation. Control group: 65% mortality; nano-selenium (SeNPs): 35%; vitamin E (VE): 45%; SeNPs + VE combination: 30%. The combination treatment provided the lowest mortality rate. Reproduced from Bazina et al., BMC Vet Res 2025, 21, 50 under CC BY 4.0 license [40].

Cumulative mortality after experimental *A. flavus* challenge provides a direct, easy-to-interpret endpoint of fungal virulence and therapeutic efficacy. In Nile tilapia challenged intraperitoneally with *A. flavus*, Bazina et al. reported that unsupplemented fish reached 65% cumulative mortality by day 15, whereas fish receiving dietary nano-selenium (SeNPs), vitamin E (VE), or their combination showed substantially lower mortality rates (35%, 45%, and 30%, respectively), with the combination treatment providing the best protection (Figure 13) [40]. These two independent studies illustrate a consistent pattern across nutritional interventions: although *A. flavus* challenge in tilapia reliably produces high mortality in unsupplemented controls, dietary antioxidants and phytogenic immunostimulants can meaningfully reduce the mortality burden, opening a practical and sustainable avenue for aspergillosis control in aquaculture.

**Figure 14:**
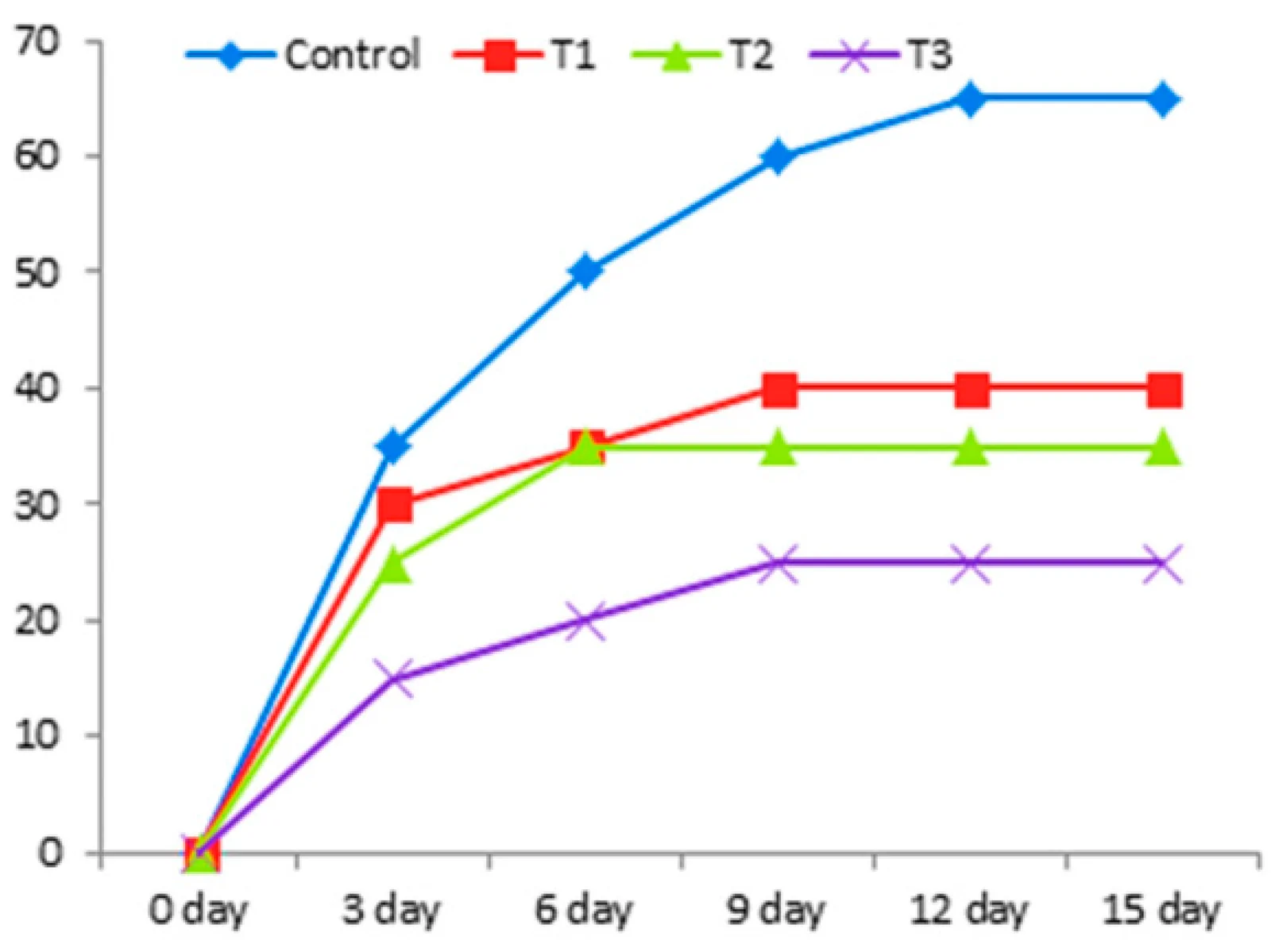
Cumulative mortality (%) of Nile tilapia (*O. niloticus*) after challenge with *Aspergillus flavus* for 15 days, in fish fed diets containing 0 (Control), 1% (T1), 2% (T2), and 3% (T3) *Phyllanthus emblica* powder. Mortality rates were significantly reduced in treatment groups compared to the control: 65% (Control), 40% (T1), 35% (T2), and 25% (T3). The 3% inclusion provided the best protection. Reproduced from Jastaniah & Albaqami, Scientific Reports 2025, 15, 11226 under CC BY 4.0 license [38].

A parallel challenge model by Jastaniah and Albaqami, using the same 65% control mortality baseline, demonstrated that dietary inclusion of Phyllanthus emblica powder reduced mortality in a dose-dependent fashion: 40% at 1% inclusion (T1), 35% at 2% (T2), and 25% at 3% (T3), with the 3% group providing the strongest survival benefit (Figure 14) [38].

### 3.4. Bioaccumulation and Food Safety

Aflatoxins and their hydroxylated metabolites can persist in fish tissues and be transferred to human consumers through fillet, liver, or roe consumption [25,67]. The exact residue levels depend on species, exposure duration, dietary concentration, and tissue type, but several studies have documented AFB1 residues in edible fillets at concentrations that, while usually below acute toxicity thresholds for humans, are relevant for chronic dietary exposure assessments [50,67]. This creates a One Health concern that links aquaculture management, veterinary medicine, and public health, and that justifies the inclusion of aflatoxin monitoring within national aquafeed surveillance programs.

### 3.5. Mitigation Strategies

Strategies to mitigate aflatoxin contamination of aquafeeds fall into four categories. The first is prevention at source, through good agricultural and storage practices: harvesting at appropriate moisture levels, rapid drying, storage below 14% moisture and 25 °C, rodent and insect exclusion, and regular monitoring [25]. The second is physical adsorption in feed or in the digestive tract, using clay minerals (bentonite, montmorillonite), synthetic polymers, activated charcoal, or engineered nanomaterials; Hassaan et al. demonstrated that nano-zeolite effectively mitigates AFB1 toxicity in Nile tilapia, with measurable improvements in growth, antioxidant status, DNA damage markers, and reduction of AFB1 tissue residues [67]. More recently, Zahran et al. evaluated a novel anti-mycotoxin agent specific for aquatic species, combining bentonite, sepiolite, and orange peel meal from *Citrus sinensis*; the formulation counteracted AFB1-induced growth suppression, immune impairment, and oxidative stress in Nile tilapia and, notably, reduced AFB1 residues in the musculature by approximately 99.6% after 6 weeks, providing a promising integrated mitigation tool with direct food-safety benefits [68]. The third is chemical or enzymatic degradation: certain bacteria and fungi produce enzymes capable of degrading aflatoxins or related mycotoxins into less toxic products; illustratively, Abrunhosa and Venâncio isolated and purified an enzyme from *A. niger* itself that hydrolyses ochratoxin A, demonstrating that the same genus that produces toxins also harbors species capable of detoxifying them [69]. Complementary biological approaches include the dietary use of microalgae such as *Spirulina platensis*, which has been shown to mitigate aflatoxin B1-induced toxicity in Nile tilapia (*Oreochromis niloticus*) by reducing serum hepatic enzymes (ALT, AST, ALP), restoring total protein and albumin, and improving antioxidative function [70]. The fourth is biological mitigation through dietary probiotics, plant extracts, and antioxidants that attenuate aflatoxin-induced damage rather than removing the toxin itself [37,38,71,72].

In practice, however, these mitigation measures are difficult to apply in small-scale and low-resource aquaculture, which dominates production in many tropical regions. Hot and humid climates accelerate fungal growth and aflatoxin formation during the post-harvest handling and on-farm storage of feed ingredients, while controlled drying, cool storage and rodent or insect exclusion are often unavailable to small producers. Routine monitoring is further constrained by the cost and limited accessibility of analytical facilities: confirmatory methods such as HPLC require centralized laboratories, and even rapid ELISA or lateral-flow test kits represent a recurring expense that is rarely sustainable at the smallholder level. As a result, much of the aflatoxin burden in these settings goes undetected, and practical, low-cost screening tools together with improved on-farm storage remain priorities for protecting both fish health and food safety.

## 4. *Aspergillus* as a Beneficial Agent: From Probiotic to Industrial Fermenter

### 4.1. Why Not All Aspergilli Are Harmful: The Strain and Species Question

**Figure 15:**
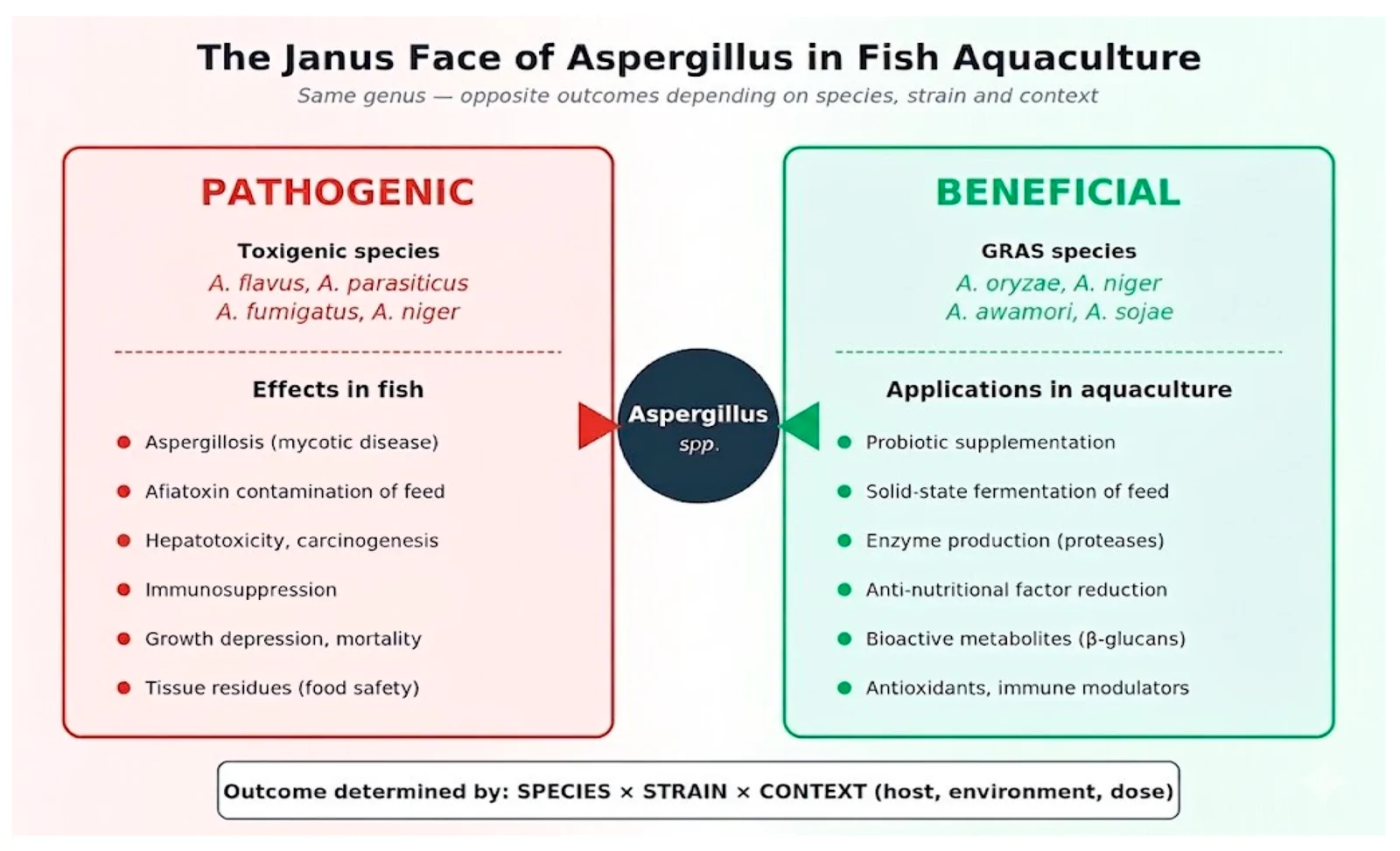
The Janus face of *Aspergillus* in fish aquaculture, a conceptual overview of the dual nature of the genus. On the pathogenic side (**left**): toxigenic species (*A. flavus*, *A. parasiticus*, *A. fumigatus*) cause aspergillosis, aflatoxin contamination, hepatotoxicity, immunosuppression, and food safety concerns. On the beneficial side (**right**): GRAS species (*A. oryzae*, *A. niger*, *A. awamori*, *A. sojae*) are used as probiotics, in solid-state fermentation, for enzyme production, anti-nutritional factor reduction, and as sources of bioactive metabolites and marine antimicrobials. The outcome depends on three factors: SPECIES × STRAIN × CONTEXT (host, environment, dose).

A key point of mycology is often underappreciated outside the field: the distance between a pathogenic, toxigenic *Aspergillus* and a beneficial, food-grade *Aspergillus* can be smaller than that between species; it can lie at the level of strains and even of gene clusters within strains. *A. oryzae*, the koji mold used for more than a thousand years to produce sake, soy sauce, and miso, is a domesticated, genetically distinct descendant of wild *A. flavus* lineages; it has effectively lost the functional aflatoxin biosynthetic cluster during centuries of selection on fermentation substrates [73]. Similarly, industrial strains of *A. niger* used for citric acid, enzyme, and feed-grade fermentation are selected and tested for the absence of ochratoxin A and fumonisin production [5,20]. The U.S. FDA therefore lists specific *A. oryzae* and *A. niger* strains as GRAS, while other strains of the same morphospecies remain of concern. Crucially, GRAS designation applies to defined, individually authenticated strains and does not imply that an entire species, or any given aquaculture application, is safe by default; strain authentication, mycotoxin screening and case-by-case safety validation therefore remain prerequisites before any *Aspergillus* strain is used in fish feed. Moreover, GRAS status was granted for defined uses in human food fermentation and industrial enzyme production, and these contexts are not equivalent to the incorporation of live or inactivated fungal biomass into aquafeed; GRAS designation therefore cannot be directly extrapolated to aquaculture and is not, on its own, sufficient to establish the safety of an *Aspergillus* preparation for fish-feed use, which requires dedicated species- and strain-specific validation. This duality, the same genus harboring both dangerous and useful members, can be summarized conceptually as the “Janus face” of *Aspergillus* in fish aquaculture (Figure 15). On the pathogenic side stand the toxigenic species (*A. flavus*, *A. parasiticus*, *A. fumigatus*) and their consequences for fish: aspergillosis, aflatoxin contamination of feed, hepatotoxicity, immunosuppression, and food safety concerns. On the beneficial side stand the GRAS species (*A. oryzae*, *A. niger*, *A. awamori*, *A. sojae*) and their applications: probiotic supplementation, solid-state fermentation, enzyme production, anti-nutritional factor reduction, and production of bioactive metabolites including marine antimicrobials. The final outcome in any given aquaculture context depends on three interacting factors: the *Aspergillus* species, the specific strain used, and the context of exposure (host, environment, dose).

The following subsections deal exclusively with the beneficial side, that is, with the use of well-characterized, non-toxigenic *Aspergillus* strains as probiotics, fermentation starters, enzyme producers, and sources of bioactive metabolites in fish aquaculture. The coherent narrative is not that *Aspergillus* is harmful or beneficial, but that its effect in fish farming is species-, strain-, and context-dependent, and that modern aquaculture increasingly relies on the careful selection of beneficial strains from a genus whose reputation has historically been dominated by its harmful members [5,20].

### 4.2. Aspergillus as a Direct Probiotic in Fish Diets

A growing body of experimental work demonstrates that dietary supplementation with live or heat-inactivated *Aspergillus* preparations improves growth, immune responses, and disease resistance in several finfish species. The mechanisms invoked include modulation of the gut microbiota, enhancement of digestive enzyme activity, immunomodulation through β-glucans and other cell-wall components, production of antimicrobial secondary metabolites, and competitive exclusion of potential pathogens [74,75].

**Figure 16:**
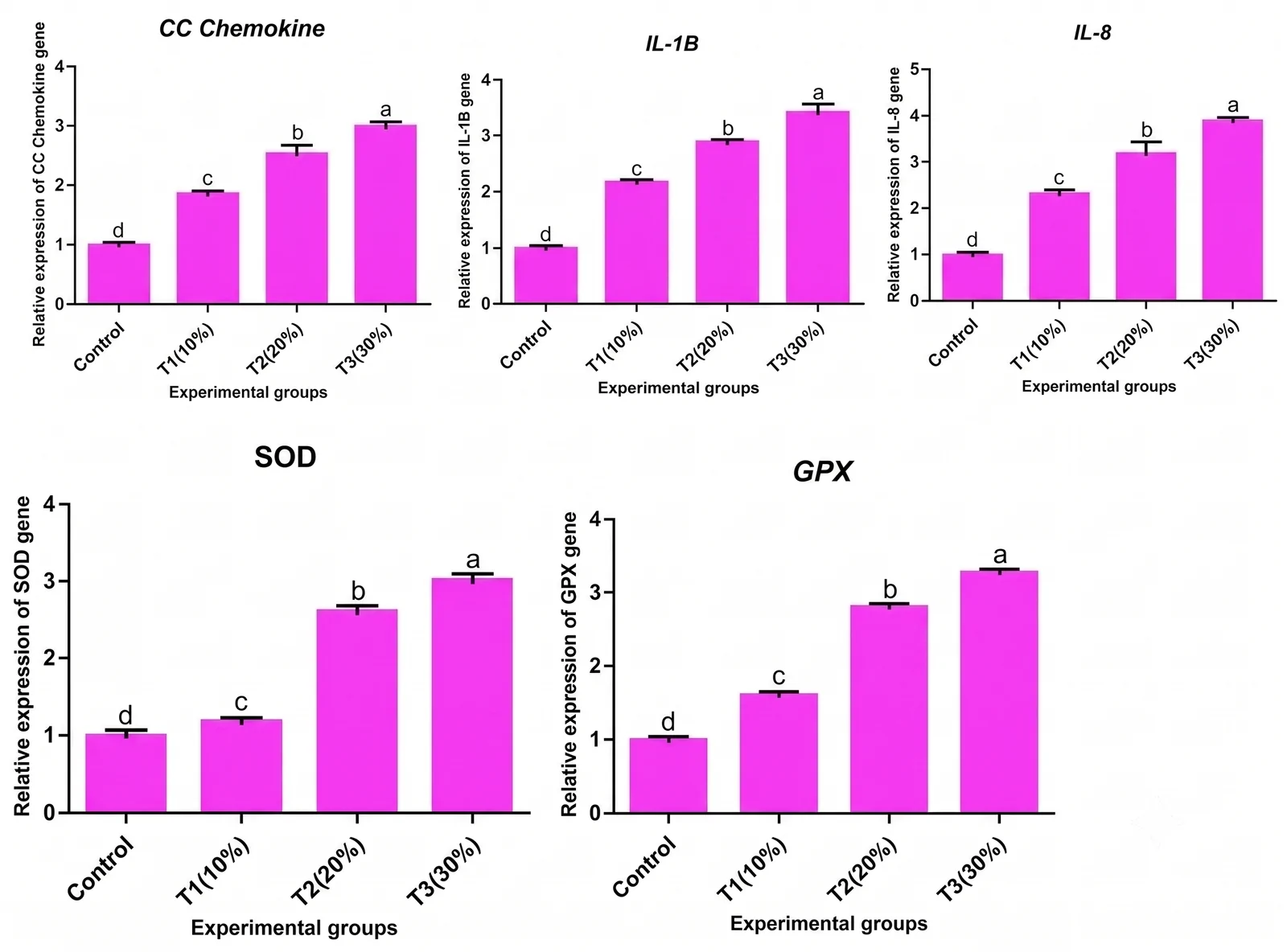
Transcriptomic profile of immunity- and antioxidant-related genes in Nile tilapia (*O. niloticus*) fed diets containing 0 (Control), 1% (T1), 2% (T2), and 3% (T3) levels of *Phyllanthus emblica* powder. Five key genes were analyzed: CC chemokine, IL-1β, and IL-8 (immune-related cytokines) and SOD and GPx (antioxidant enzymes). All genes were significantly upregulated in a dose-dependent manner (*p* < 0.05), with the highest expression observed in the 3% treatment group. Different letters above bars (a, b, c, d) indicate statistically significant differences. Reproduced from Jastaniah & Albaqami, Scientific Reports 2025, 15, 11226 under CC BY 4.0 license [38].

Jastaniah and Albaqami provided detailed transcriptomic evidence of the immunomodulatory effects of dietary phytogenic supplementation in Nile tilapia, studying the expression of five key genes: CC chemokine, IL-1β, IL-8 (immune-related cytokines), and SOD and GPx (antioxidant enzymes), in fish fed *Phyllanthus emblica* powder at 1%, 2%, and 3% dietary inclusion (Figure 16) [38]. All five genes were upregulated in a dose-dependent manner (*p* < 0.05), with the strongest response observed in the 3% inclusion group. The coordinated upregulation of both pro-inflammatory cytokines and antioxidant enzymes indicates that the protective effect of *P. emblica* against *A. flavus* challenge, documented histologically (Figure 9 and Figure 10) and in terms of mortality (Figure 14), is supported by a coherent transcriptomic reprogramming of the innate immune and antioxidant machinery of the fish. Such molecular signatures complement the histological endpoints and provide a mechanistic rationale for the use of plant polyphenols as functional feed additives against fungal challenge in aquaculture.

Beyond plant-derived extracts, dietary *Aspergillus* itself has been extensively studied as a probiotic in tilapia and other species. Dawood et al. reported that dietary inclusion of *A. oryzae* in Nile tilapia diets improved oxidative status, modulated heat shock protein expression, and upregulated immune-related genes under hypoxia challenge [15]. In a follow-up synbiotic study, the same group combined *A. oryzae* with β-glucan and documented complementary effects on growth and oxidative and immune responses in Nile tilapia [76]. Shukry et al. demonstrated that dietary *A. oryzae* modulates serum biochemistry, immune responses, oxidative stress, and transcription of HSP70 and cytokine genes in Nile tilapia exposed to salinity stress [16]. Iwashita et al. showed that a dietary combination of *Bacillus subtilis*, *Saccharomyces cerevisiae*, and *A. oryzae* enhanced immunity and disease resistance against *Aeromonas hydrophila* and *Streptococcus iniae* infection in juvenile tilapia [17]. Jasim et al. reported probiotic effects of *A. niger* on growth, immunity, hematology, intestinal fungal load, and digestive enzymes of the common carp (*Cyprinus carpio*) [18]. In common carp, Hedayati documented growth and hemato-immunological improvements in response to fermented *A. oryzae* [77], and Naser et al. showed that dietary *A. oryzae* improved productive traits of common carp under Iraqi rearing conditions [78]. Dawood et al. demonstrated that *Aspergillus oryzae*-fermented date palm seed meal, included at up to 200 g/kg in Nile tilapia diets, dose-dependently improved growth performance (final weight, weight gain, specific growth rate), enhanced digestive enzyme activities (lipase, amylase, protease), increased hematological parameters (Hb, RBCs, WBCs), and improved intestinal architecture (villus length and goblet cell numbers), thereby valorizing agricultural waste as a sustainable *Aspergillus*-fermented feed ingredient [79]. For comparison, similar methodological and regulatory standards have been applied to bacterial probiotics in tilapia, including dietary *Bacillus subtilis* [80], multi-strain water probiotics [81], and *Bacillus rugosus* NM007 [56], all of which exemplify the rigorous evaluation now expected for any probiotic candidate. *Aspergillus oryzae* itself has been comprehensively reviewed for its postbiotic potential, including non-viable cells/cell-fragments, enzymes, extracellular polymeric substances, and bioactive metabolites with documented benefits in Nile tilapia (immune system enhancement, oxidative stress reduction, growth promotion, and disease resistance) [82]. Complementary dietary approaches, such as organic acid blends in red tilapia broodstock [83], further illustrate the expanding toolbox of functional additives for sustainable tilapia aquaculture. Taken together, these studies indicate that the benefits of dietary *Aspergillus* are not strain-specific anecdotes but a reproducible pattern: across tilapia and other species, *A. oryzae* supplementation consistently enhances antioxidant capacity, immune gene expression and stress resistance, most likely through a combination of cell-wall β-glucan immunostimulation and improved nutrient availability. Table 4 summarizes representative probiotic studies.

The beneficial or detrimental outcome of an *Aspergillus* supplement depends strongly on dose and delivery. Reported effective inclusion levels vary with strain, fish species and rearing conditions, and higher concentrations do not necessarily give better results: an excessive fungal load can shift the balance from beneficial to detrimental, disturbing the resident gut microbiota, increasing competition for nutrients and oxygen, and raising the metabolic and immune cost to the host. Outcomes therefore reflect not only the supplemented strain but the total microbial load and the balance between beneficial and potentially pathogenic community members, including synergistic and antagonistic interactions, competition for nutrients, and chemical (quorum-sensing) communication. Dose optimization and monitoring of the wider microbial community are thus essential when *Aspergillus* is used as a probiotic.

### 4.3. Solid-State Fermentation of Plant Ingredients by Aspergillus

**Figure 17:**
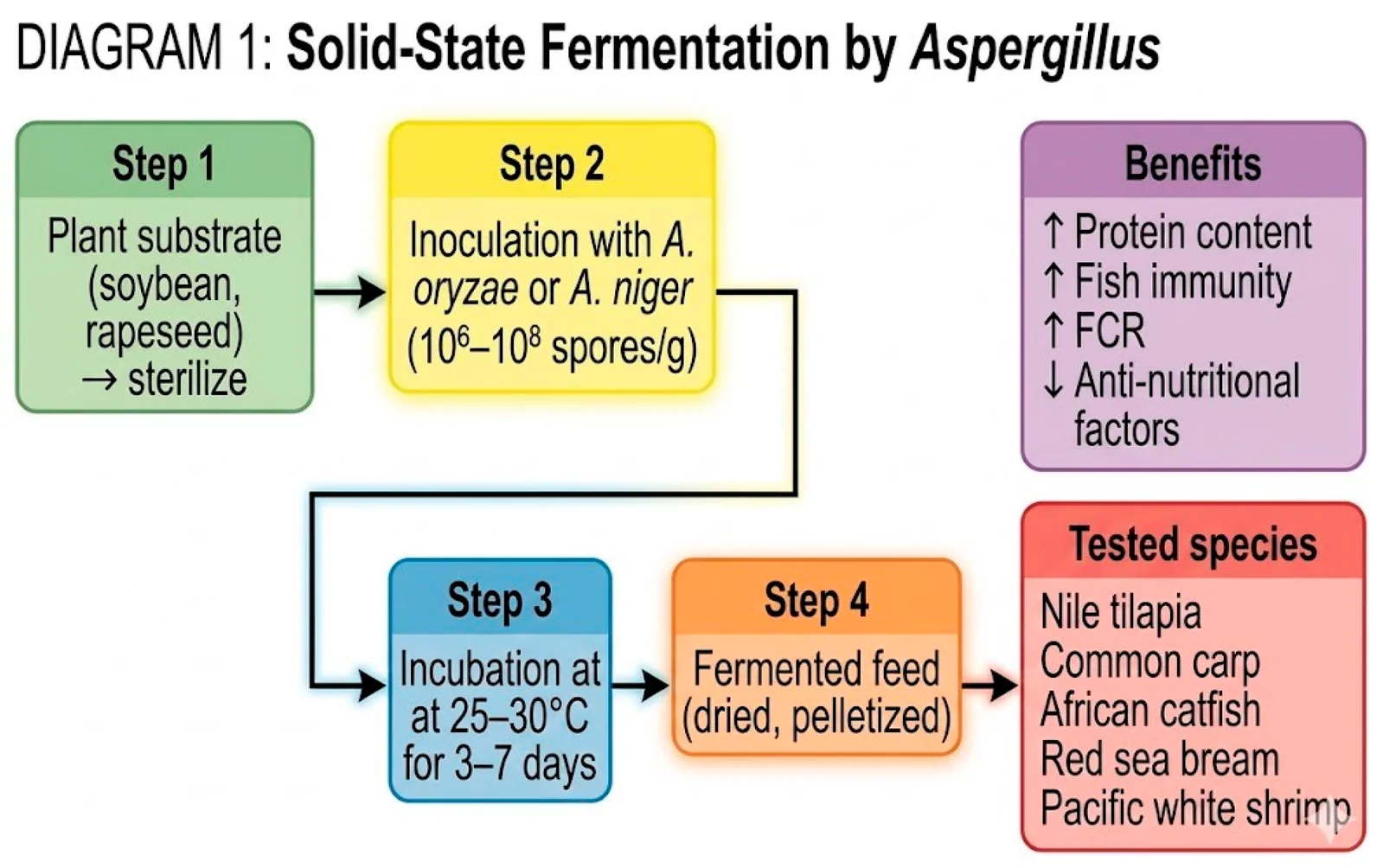
Conceptual schematic of solid-state fermentation (SSF) of plant feed ingredients by *Aspergillus oryzae* or *A. niger*. Four steps: (1) sterilization of plant substrate (soybean, rapeseed, olive cake, palm kernel); (2) inoculation with a controlled spore suspension (10^6^–10^8^ spores/g); (3) incubation at 25–30 °C for 3–7 days under controlled humidity; (4) drying and palletization as fishmeal substitute. During fermentation, crude protein, free amino acids, and digestive enzymes increase, while phytates, glucosinolates, tannins, and trypsin inhibitors decrease. Documented benefits in vivo include improved growth, FCR, gut histology, immunity, and disease resistance in tilapia, carp, sea bream, and shrimp.

Solid-state fermentation (SSF) of plant-based feed ingredients is one of the most economically valuable uses of *Aspergillus* in aquaculture [5,20]. The main idea is that plant products such as soybean, rapeseed, cottonseed, olive cake, and rice bran have antinutritional factors (tannins, phytates, glucosinolates, non-starch polysaccharides, trypsin inhibitors), which restrict their application in fish feed [5,20]. These antinutrients can be hydrolyzed by *Aspergillus* species, which have a rich repertoire of hydrolytic and oxidative enzymes, which can break them down during SSF and at the same time raise the amount of crude protein, lessen fibers, and release bioactive peptides and organic acids [5,9,10,11,20,84]. The process comprises four sequential steps, summarized in Figure 17: (1) sterilization of the plant substrate (typically soybean meal, rapeseed, olive cake, or palm kernel) by autoclaving or steam; (2) inoculation with a controlled spore suspension of *A. oryzae* or *A. niger* at 10^6^–10^8^ spores/g; (3) incubation at 25–30 °C for 3–7 days under controlled humidity; and (4) drying and palletization of the fermented product for direct use as a fishmeal substitute [10,11,84]. Biochemical changes during fermentation include a dramatic increase in crude protein, free amino acids, bioactive peptides and a corresponding decrease in phytates, glucosinolates, tannins and trypsin inhibitors, the combination of which has been found to account for the consistent positive in vivo effects (improved growth, feed conversion ratio, gut histology, immunity and disease resistance) observed across tilapia, common carp, red sea bream, and Pacific white shrimp (Figure 17).

Dossou et al. demonstrated that replacing part of fishmeal with *A. oryzae*-fermented rapeseed meal (rapeseed meal-Koji, RM-Koji) significantly improved growth performance, blood health, antioxidant status, and immune response in red sea bream (*Pagrus major*) [10]. Shi et al. demonstrated that SSF of rapeseed cake with *A. niger* degraded glucosinolates and improved the nutritional value of the ingredient [84]. Dayal et al. found that *A. niger*-fermented plant protein mix can partially replace fishmeal in the diet of *Penaeus vannamei*, which has implications in the wider formulation of aquafeeds [9]. Ismail et al. applied the concept of fermentation to the olive cake and showed positive outcomes in gut immune gene expression, histomorphometry, and haemato-immunological index in Nile tilapia fed *A. oryzae*-fermented olive cake [11]. Mechanistically, these studies converge on a single principle: by secreting hydrolytic and detoxifying enzymes during fermentation, optimally chosen strains of *Aspergillus* may transform low-value, high-antinutrient plant by-products into high-value, functional feed components, enabling the move to a more circular and lower-impact aquaculture feed system. Importantly, because even non-toxigenic *Aspergillus* strains can express otherwise silent secondary-metabolite gene clusters under fermentation conditions, the safe use of solid-state fermentation requires defined quality-control safeguards, including verified strain stability across production batches, routine mycotoxin testing of the fermented product, and, ideally, metabolomic safety screening to exclude undesirable secondary metabolites before the ingredient enters the feed chain. The fermentation parameters cited above (10^6^–10^8^ spores/g, 25–30 °C and 3–7 days) should be read as general starting ranges rather than universal optima, since the ideal inoculum density, temperature and duration are strongly substrate- and strain-dependent and must be optimized for each substrate–*Aspergillus* combination. The in-vivo improvements reported with these fermented ingredients (growth, antioxidant capacity, immune gene expression and intestinal morphology) likely arise from two complementary mechanisms: the reduction of antinutritional factors with the resulting gain in nutrient digestibility, and the direct immunomodulatory action of fungal cell-wall components (notably β-glucans), secreted enzymes and fungal metabolites; disentangling their relative contributions remains an open question (Table 5).

**Table 5:** Reported solid-state fermentation conditions for representative substrate–*Aspergillus* combinations in aquafeed studies.

Substrate	*Aspergillus* Species	Inoculum	Temperature	Duration	Fish Species	Ref.
Rapeseed meal (koji)	*A. oryzae*	10^8^ g^−1^ starter (4 g/kg)	30 °C then 37 °C (95% RH)	~2 days	Red sea bream	[10]
Olive cake	*A. oryzae*	10^8^ g^−1^	30 °C	2 days	*Nile tilapia*	[11]
Plant-protein mix (soybean-based)	*A. niger*	10^7^ spores/mL	35 ± 1 °C	3 days	*Penaeus vannamei* (shrimp)	[9]

### 4.4. Industrial Enzymes from Aspergillus for Aquaculture

The species of *Aspergillus* lead in the market of industrial enzymes. Specifically relevant enzymes to aquaculture are phytases (liberating phosphorus in plant phytate and reducing phosphorus pollution of fish farms), proteases, α-amylases, cellulases, xylanases, α-glucanases, tannases, and lipases [5,20]. A large number of commercial enzymes currently used in aquafeeds are the result of fermentation of *A. niger*, *A. oryzae*, or *A. awamori* isolates chosen on the basis of large yield and non-toxicity of their fermentates. The nutrient digestibility in their dietary inclusion is generally enhanced, the ratio of feed conversion is reduced, and the environmental impact of fish farming is minimized, which is a quietly revolutionary addition of *Aspergillus* to aquaculture sustainability.

### 4.5. Bioactive Secondary Metabolites

In addition to enzymes, non-toxigenic strains of *Aspergillus* generate an abundant and largely unexploited repertoire of bioactive secondary metabolites with possible use in the health management of aquaculture. *Aspergillus* cell walls contain β-glucans that are identified as immunostimulants in fish that boost non-specific immunity and disease resistance [75]. *A. niger* extracts and some *A. flavus* variants have been studied as antimicrobial and antitumor agents in vitro, and they have also shown complex structure-activity relationships [85]. Such fungal bioactives are complementary to the larger trend of phytobiotic and natural-origin feed additives in aquaculture, which has recently been reviewed by Kalaiselvan et al., who systematically synthesized the sources, mode of action, effects, administration and bioavailability of phytobiotics in fish encompassing diverse bioactive classes such as essential oils, alkaloids, phenolic compounds, carotenoids, saponins, terpenoids, flavonoids, and glycosides, many of which exhibit antimicrobial and antifungal properties relevant to the management of *Aspergillus* and aflatoxin challenges in tilapia aquaculture [29]. The most promising literature, in particular, is the rapidly growing body of research on marine-derived *Aspergillus* spp., which have produced many new antimicrobial compounds with activity against fish pathogens including *Vibrio* spp., *Streptococcus iniae*, and *Edwardsiella tarda* [27,28]. Though the chemistry of these metabolites is not discussed in detail within the scope of this review, they demonstrate a larger argument: the same genus which is the source of some of the most toxic natural products (aflatoxins) is the source of some of the most promising antimicrobial candidates in aquaculture.

### 4.6. Aspergillus in Bioremediation of Aquaculture Effluents

A fourth and emerging beneficial role of *Aspergillus* in aquaculture concerns effluent management and bioremediation. Onomu and Okuthe, reviewing the application of fungi and their secondary metabolites in aquaculture, highlighted the use of *Aspergillus* spp. for flocculation of suspended solids, degradation of antibiotic residues and other xenobiotics, and reduction of nitrogen and phosphorus loads in recirculating aquaculture system (RAS) effluents [5]. This role complements the feed-side applications and positions *Aspergillus* within an integrated vision of circular, low-impact aquaculture where the same microbial genus contributes at several points of the production chain.

### 4.7. Marine-Derived Aspergillus and the One Health Perspective

A phylogenetically and metabolically diversified *Aspergillus* subpopulation, which is more extensively explored as a biotechnological resource, is found in marine ecosystems [27,28,48]. Li et al. investigated 98 antimicrobial compounds isolated from marine *Aspergillus* between 2021 and 2023, with sponges (23.3%) and corals (16.7%) as the most frequent ecological sources and emphasized their applicability as a source of lead molecules in combating fish pathogens. Among notable examples are asperalins from *A. alabamensis* that exhibit strong anti-*Streptococcus iniae* (MIC = 2.2 μM) and anti-*Edwardsiella ictaluri* (MIC = 10.9 μM) potencies in addition to trypacidin from *A. fumigatus* that reveals anti-*Vibrio harveyi* activity equivalent to streptomycin [27]. Wang et al. comprehensively reviewed 337 antibacterial natural products (including 145 new compounds) isolated from marine-derived *Aspergillus* species over 14 years (2010–2024), with multiple compounds showing activity against fish pathogens of direct aquaculture relevance, including aspergixanthones I–K from *Aspergillus* sp. ZA-01 active against three pathogenic *Vibrio* species (*V. parahemolyticus*, *V. anguillarum*, and *V. alginolyticus*) with MIC values of 1.56 to 25.0 μM, supporting the use of marine *Aspergillus* as a One Health resource for sustainable aquaculture [28]. These findings dovetail with broader investigations of marine fungi as pathogens and as sources of bioactive molecules [48], and support a One Health framework in which aquaculture, ocean biodiversity, and public health are studied and managed together.

## 5. Integrative Discussion: Friend or Foe?

### 5.1. Synthesizing the Paradox

The analyzed evidence confirms a consistent integrative approach: the role of *Aspergillus* in fish aquaculture is established simultaneously by (i) the species, (ii) the strain, and (iii) the context of exposure. At the species level, *A. fumigatus*, *A. flavus*, and *A. parasiticus* are biased towards the pathogenic, toxigenic side [7,26,34,42], whereas *A. oryzae*, *A. niger* and *A. awamori* lineages are biased towards the beneficial side [10,15,17,79]. On the strain level, there are both toxigenic and non-toxigenic isolates of *A. flavus* and *A. niger* and their behavior cannot be predicted based on morphology alone [73]. At the context level, even a potentially useful strain would be a risk, when used on immunosuppressed fish, in low water quality, or on substrates which encourage toxin generation during storage [25,65]. On the other hand, the effect of wild-type *Aspergillus* pressure on the environment can be absorbed by good biosecurity, and in part offset by the competitive influence of deliberately released probiotic strains [51,52].

This three-level system (species × strain × context) is not just a theoretical convenience. It has direct operational implications: regulatory frameworks, commercial probiotic certification schemes, and on-farm management all need to consider *Aspergillus* at the strain level rather than treating the genus as a monolithic entity [51,73]. It follows that neither safety nor pathogenicity should be inferred for an entire *Aspergillus* species from evidence generated with one or a few strains; species-level statements throughout this review should be read with this limitation in mind.

### 5.2. Current Knowledge Gaps

There are a number of gaps in the existing literature. To begin with, identification of *Aspergillus* isolates in diseased fish at the species level has been inconsistent and a large number of historical records are likely to contain misidentified or cryptic species. Epidemiological picture would be improved significantly by systematic molecular redescription of archived isolates, and routine use of ITS/β-tubulin/calmodulin sequencing in diagnostic laboratories. Second, mycobiomes of healthy and diseased fish remain largely unexplored, and metagenomic and amplicon-based fungal (as opposed to bacterial) studies are limited. Third, the dose-response maximization of *Aspergillus* probiotics in fish species, life stages, and rearing conditions remains incomplete; the vast majority of published works involve a single dose in comparison to an untreated control without an in-depth examination of the response curve shape. Fourth, there is limited long-term safety information, especially potential residues of *Aspergillus*-derived metabolites in edible tissues, to support the use of probiotics. Fifth, in the scientific literature, commercial probiotic products with *A. oryzae* or *A. niger* composition are seldom described at the strain level, thus making independent reproducibility and risk assessment challenging.

Antimicrobial resistance is an increasingly important consideration for both pathogenic and beneficial strains. Azole resistance in *Aspergillus fumigatus* is now a well-documented and emerging problem, driven not only by clinical azole exposure but also by environmental selection through azole fungicides used in agriculture [86]; azole-non-wildtype *A. fumigatus* and *A. flavus* isolates have also been reported from a range of animals and their direct environment [87]. Because therapeutic options for fish aspergillosis are already limited, such resistance would further constrain treatment, while beneficial fungal strains and co-administered bacterial probiotics could act as reservoirs of transferable resistance determinants. Screening candidate probiotic strains for acquired resistance genes, and avoiding potential resistance reservoirs, should therefore be an explicit part of safety assessment before use in aquafeed.

### 5.3. Challenges and Future Perspectives

In the future, a number of research directions seem to be especially promising. Whole-genome sequencing, and phylogenomic study of *Aspergillus* strains utilized in aquaculture would enable resilient classification between toxigenic and non-toxigenic lineages and aid evidence-based certification of commercial probiotics. Specific molecular strategies, such as CRISPR-based inactivation of aflatoxin biosynthesis gene clusters, will potentially create safer industrial strains, but regulatory and public acceptance challenges will have to be carefully walked. Multi-omics analysis (transcriptomics, metabolomics, gut microbiome profiling) correlating particular *Aspergillus* strains with a given fish phenotype would help speed the rational design of functional feeds. The probable effect of climate change on the distribution and toxigenicity of *Aspergillus* in the aquaculture supply chains is worth systematic evaluation, particularly in tropical and subtropical areas where the aquaculture development is concentrated. Lastly, the standardization of the experimental protocols such as dose reporting, challenge models, endpoint choice and duration would significantly enhance comparability across studies and would allow synthesis in future reviews through meta-analysis.

### 5.4. Practical Recommendations for Fish Farmers and Veterinarians

**Figure 18:**
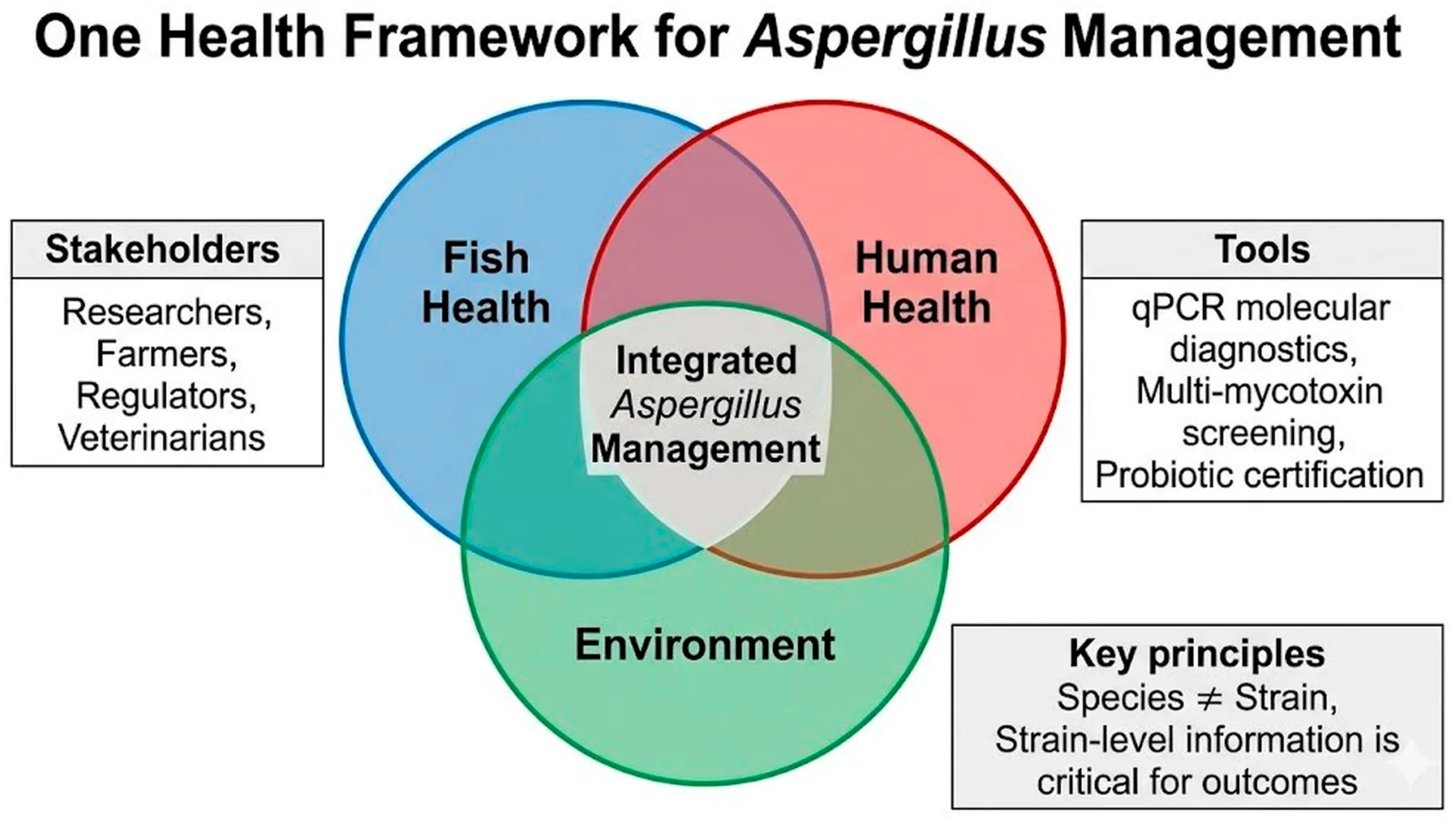
One Health framework for *Aspergillus* management in aquaculture. The integrated management of *Aspergillus* sits at the intersection of fish health (animal welfare, veterinary care), human health (food safety, consumer protection), and the environment (sustainable feed, waste reduction). Key stakeholders (fish veterinarians, feed manufacturers, aquaculturists, mycologists, food safety regulators) must collaborate using modern tools (molecular diagnostics, multi-mycotoxin screening, strain-specific products, probiotic certification, climate-resilient storage). Two key principles emerge: (i) species ≠ strain, with strain-level information critical for risk assessments; and (ii) the same genus offers both threats and opportunities: sustainable aquaculture requires managing the risks while harnessing the benefits.

Turning the scientific evidence into practice, one can provide several practical suggestions. Feed storage must be cool, dry, well-ventilated, rodent and insect-free, and designed to have short turnover cycles. Periodic laboratory evaluation of aflatoxin levels should be supplemented by regular sensory analysis of feed (discoloration, odors, and clumping) especially with plant-based formulations. The purchase of probiotic products using *Aspergillus* should be done only with suppliers who can produce documentation of strain identity, lack of mycotoxin formation, and efficacy information in the target species. On-farm biosecurity must involve quarantine of new stock, disinfection of nets and equipment and regular elimination of dead fish to reduce environmental spore loads. In cases of suspicion of clinical aspergillosis, laboratory culture and, preferably, molecular identification are highly recommended, since species-level data are used to inform both immediate management (e.g., feed batch disposal) and preventive (long-term) actions. Lastly, integrated strategies including good water quality, balanced nutrition, bacterial probiotics, dietary antioxidants, and, where available, plant-based antifungals have been repeatedly shown to be more effective than single-agent intervention based on experimental challenge studies [37,88]. Taken together, these practical measures fit into a broader One Health framework where the integrated management of *Aspergillus* sits at the intersection of fish health (animal welfare, veterinary care), human health (food safety, consumer protection), and the environment (sustainable feed, waste reduction). Achieving this balance requires the collaboration of fish veterinarians, feed manufacturers, aquaculturists, mycologists, and food safety regulators, supported by modern tools such as molecular diagnostics, multi-mycotoxin screening, strain-specific probiotic certification, and climate-resilient storage (Figure 18).

In practical terms, *Aspergillus* is beneficial when a non-toxigenic, strain-authenticated species (typically *A. oryzae* or *A. niger*) is used at an optimized dose as a probiotic or fermentation agent under controlled conditions, and detrimental when toxigenic species (such as *A. flavus*, *A. parasiticus* or *A. fumigatus*) contaminate feed or infect fish, especially under poor storage, high stocking density, or immunosuppression. The decisive factors are species and strain identity, verified absence of toxigenicity, dose, and rearing conditions, rather than the genus itself.

When deciding whether to use an additive derived from an *Aspergillus* strain—whether a probiotic, a fermented ingredient, or a metabolite preparation—a structured safety assessment is advisable. Key steps are: (i) unambiguous species- and strain-level identification by molecular methods; (ii) verification that the strain lacks functional mycotoxin (e.g., aflatoxin, ochratoxin) biosynthetic pathways, confirmed by genomic screening and analytical testing of the product; (iii) screening for acquired antimicrobial-resistance determinants; (iv) characterization of the metabolite profile of the final product to exclude undesirable secondary metabolites; and (v) dose-finding and stability testing under production conditions. Only strains and products passing all of these checks should enter the feed chain, and batch-level mycotoxin and metabolite testing should be maintained thereafter.

Reliable detection of aflatoxins and other mycotoxins in aquafeed and fish tissues underpins these safety measures. Rapid screening methods such as enzyme-linked immunosorbent assays (ELISA) and lateral-flow immunochromatographic strips are inexpensive and suitable for on-farm or feed-mill use, but are only semi-quantitative and can suffer from matrix interference. Quantitative confirmation relies on chromatographic methods: high-performance liquid chromatography with fluorescence detection (HPLC-FLD), usually after immunoaffinity clean-up, and liquid chromatography coupled to tandem mass spectrometry (LC-MS/MS), which allows sensitive, specific multi-mycotoxin quantification; thin-layer chromatography (TLC) remains a low-cost qualitative option. A tiered strategy—rapid immunoassay screening followed by chromatographic confirmation of positive samples—gives the best balance of cost, speed and accuracy for routine aquaculture monitoring (Table 6).

The main strength of this review is its integrative, One Health scope, combining pathogenic, toxicological and biotechnological evidence for *Aspergillus* in a single finfish-focused synthesis, supported by comparative tables. Its limitations reflect those of the underlying literature: species-level identification is inconsistent across studies, many probiotic and fermentation trials use single doses and short durations, strain-level data are often missing, and direct aquaculture evidence is lacking for some mechanisms (e.g., biofilm formation). These constraints, discussed in Section 5.2, should be kept in mind when interpreting the conclusions.

## 6. Conclusions

The genus *Aspergillus* embodies, within fish aquaculture, a biological paradox that is better understood as a continuum than as a dichotomy. Certain species and strains cause aspergillosis in farmed fish and contaminate aquafeeds with some of the most hepatotoxic compounds known, while others, sometimes from the same morphospecies, serve as probiotics, fermentation agents, enzyme producers, and sources of bioactive metabolites that support sustainable, plant-protein-based aquaculture. Far from contradicting each other, these two faces of *Aspergillus* reflect the genuine biological diversity of the genus and the critical role of species identity, strain characterization, and environmental context in determining outcomes. Recent 2026 developments, including novel anti-mycotoxin formulations capable of dramatically reducing AFB1 tissue residues in Nile tilapia [68], updated reviews of mycotoxin pathways in modern plant- and insect-based feeds [22], the characterization of new probiotic candidates for tilapia aquaculture [56], and the identification of novel natural antifungal metabolites active against opportunistic fungi in farmed fish [27], collectively demonstrate that the field is advancing rapidly and that practical tools for managing both the pathogenic and beneficial faces of *Aspergillus* are becoming available.

The coherent path forward sits at the intersection of fish health, human health, and the environment, the classic One Health triangle, and requires the collaboration of fish veterinarians, nutritionists, mycologists, feed manufacturers, aquaculturists, food safety regulators, and public health officials (Figure 18). Modern tools such as molecular diagnostics (qPCR, ITS/β-tubulin sequencing), multi-mycotoxin screening platforms, strain-specific probiotic certification schemes, climate-resilient feed storage, and traceability systems can collectively support an integrated management approach. Two principles emerge as particularly important for evidence-based aquaculture practice and regulation: first, species ≠ strain, with strain-level information being critical for risk assessment because toxigenic and non-toxigenic isolates can coexist within the same morphospecies; and second, the same genus offers both threats and opportunities, and sustainable aquaculture must manage the pathogenic face while harnessing the beneficial face, rather than treating *Aspergillus* as a monolithic entity. Harnessing the beneficial potential of *Aspergillus* while containing its pathogenic and toxigenic face will therefore require an integrated One Health approach, an approach that places strain-level information at the center of management and regulation. The Janus face of *Aspergillus* is not a problem to be resolved by choosing one side over the other; it is a reality to be managed with rigor, humility, and a steady commitment to evidence.

In practical terms, these conclusions translate into distinct priorities for each stakeholder group. Fish farmers should prioritize cool, dry, well-ventilated feed storage with short turnover, routine sensory inspection of feed, rapid removal of dead fish, and quarantine and disinfection as front-line biosecurity. Feed manufacturers should source and document non-toxigenic, strain-authenticated *Aspergillus* cultures, apply mycotoxin testing and metabolomic safety screening to fermented ingredients, and provide batch-level certificates of strain identity and toxin absence. Researchers should standardize challenge models, dose reporting and endpoints, characterize commercial probiotic strains at the whole-genome level, and quantify long-term residues of *Aspergillus*-derived metabolites in edible tissues. Regulators should work towards aquaculture-specific aflatoxin limits, require strain-level certification of probiotic and fermentation products, and integrate fungal pathogens and mycotoxins into national aquafeed surveillance programs.

## Figures and Tables

**Table 1:** Representative reports of *Aspergillus* species isolated from cultured finfish.

*Aspergillus* Species	Fish Host	Country/Region	Reference
*A. flavus*	Nile tilapia (*Oreochromis niloticus*)	Egypt	[12,13]
*A. flavus*	Nile tilapia/red tilapia	Saudi Arabia/Egypt	[37,39,40]
*A*. *fumigatus* (dominant), *A. flavus*, *A. niger*, *A. terreus*	Tilapia (*Oreochromis* sp.)	Indonesia	[14]
*A. flavus, A. niger*	Silver carp (*Hypophthalmichthys molitrix*)	Pakistan	[41]
*A. niger*, *A. fumigatus*, *A. sydowii*	Freshwater fishes (*Channa*, *Clarias*)	India (Bhopal, Madhya Pradesh)	[7]
*A. niger*, *A. flavus* and others	African catfish (*Clarias gariepinus*), Nile tilapia	Egypt	[35]
*A. flavus*, *A. niger*, *A. terreus*	Farmed freshwater fish (multiple)	Egypt	[34,42]
*A. flavus*, *A. niger*	Persicaria-treated tilapia cohort	Egypt (Aswan)	[43]

**Table 3:** Representative studies reporting aflatoxin occurrence in fish feeds across regions.

Country/Region	Feed/Fish Species	Main Aflatoxins Detected	Reference
Kenya (Nyeri)	Tilapia feeds (commercial and homemade)	AFB1 (dominant)	[63]
Brazil (Rio de Janeiro)	Finished fish feeds	AFB1, FB1, OTA	[64]
Argentina	Rainbow trout (*O. mykiss*) feeds	AFs + *Fusarium* toxins	[65]
Italy/Europe	Aquafeeds, emerging mycotoxins review	AFs, ENNs, BEA	[21]
Egypt	Freshwater fish & feeds	AFB1	[42]
Multiple	Aquaculture feeds (review)	AFs, OTA, ZEN, FBs	[25,26]

**Table 4:** Representative studies on *Aspergillus* spp. as dietary probiotics or fermentation agents in finfish aquaculture.

Fish Species	*Aspergillus* Species/Form	Main Reported Effects	Reference
Nile tilapia (*O. niloticus*)	*A. oryzae*, dietary	Improved oxidative status, HSP70 & cytokine expression, hypoxia tolerance	[15]
Nile tilapia	*A. oryzae* + β-glucan (synbiotic)	Better growth, oxidative, and immune responses	[76]
Nile tilapia	*A. oryzae*, dietary	Improved immune response, HSP70 transcription, salinity stress tolerance	[16]
Nile tilapia (juveniles)	*B. subtilis* + *S. cerevisiae* + *A. oryzae*	Enhanced immunity, resistance to *A. hydrophila* and *S. iniae*	[17]
Nile tilapia	*A. oryzae*-fermented olive cake	Gut immune gene expression, histomorphometry, hematology benefits	[11]
Common carp (*C. carpio*)	*A. niger*, dietary	Growth, immunity, digestive enzymes, reduced intestinal fungal load	[18]
Common carp	Fermented *A. oryzae*	Growth and hemato-immunological improvements	[77]
Common carp	*A. oryzae*, dietary	Improved productive traits (Iraqi trial)	[78]
Red sea bream (*Pagrus major*)	*A. oryzae*-fermented rapeseed (RM-Koji)	Growth, blood health, antioxidant, immune responses	[10]
Shrimp (*P. vannamei*), context	*A. niger*-fermented plant protein mix	Fishmeal substitution potential	[9]

**Table 6:** Common methods for detecting aflatoxins and other mycotoxins in aquafeed and fish tissues.

Method	Type	Application	Notes
ELISA	Immunoassay	Rapid screening (feed, tissues)	Semi-quantitative; fast and low-cost; possible matrix interference
Lateral-flow strips	Immunoassay	On-site/field screening	Qualitative to semi-quantitative; very rapid; usually single-toxin
HPLC-FLD	Chromatography	Quantitative confirmation	Sensitive; needs immunoaffinity clean-up; few toxins per run
LC-MS/MS	Chromatography–MS	Multi-mycotoxin confirmation	High sensitivity and specificity; multi-analyte; costly instrumentation
TLC	Chromatography	Low-cost qualitative screening	Simple; lower sensitivity; largely superseded

## Data Availability

No new data were created or analyzed in this study. Data sharing is not applicable to this article.
